# Blood donor biobank and HLA imputation as a resource for HLA homozygous cells for therapeutic and research use

**DOI:** 10.1186/s13287-022-03182-7

**Published:** 2022-10-09

**Authors:** Jonna Clancy, Kati Hyvärinen, Jarmo Ritari, Tiina Wahlfors, Jukka Partanen, Satu Koskela

**Affiliations:** 1 Blood Service Biobank, Finnish Red Cross Blood Service, Helsinki, Finland; 2 R&D, Finnish Red Cross Blood Service, Helsinki, Finland; 3 Present Address: Finnish Institute for Health and Welfare, Helsinki, Finland

**Keywords:** Imputation, Major histocompatibility complex, HLA, Homozygosity, Cell therapy, Thrombocyte, Population, Biobank

## Abstract

**Background:**

Allogeneic therapeutic cells may be rejected if they express HLA alleles not found in the recipient. As finding cell donors with a full HLA match to a recipient requires vast donor pools, the use of HLA homozygous cells has been suggested as an alternative. HLA homozygous cells should be well tolerated by those who carry at least one copy of donor HLA alleles. HLA-A-B homozygotes could be valuable for HLA-matched thrombocyte products. We evaluated the feasibility of blood donor biobank and HLA imputation for the identification of potential cell donors homozygous for HLA alleles.

**Methods:**

We imputed HLA-A, -B, -C, -DRB1, -DQA1, -DQB1 and -DPB1 alleles from genotypes of 20,737 Finnish blood donors in the Blood Service Biobank. We confirmed homozygosity by sequencing HLA alleles in 30 samples and by examining 36,161 MHC-located polymorphic DNA markers.

**Results:**

Three hundred and seventeen individuals (1.5%), representing 41 different haplotypes, were found to be homozygous for HLA-A, -B, -C, -DRB1, -DQA1 and -DQB1 alleles. Ten most frequent haplotypes homozygous for HLA-A to -DQB1 were HLA-compatible with 49.5%, and three most frequent homozygotes to 30.4% of the Finnish population. Ten most frequent HLA-A-B homozygotes were compatible with 75.3%, and three most frequent haplotypes to 42.6% of the Finnish population. HLA homozygotes had a low level of heterozygosity in MHC-located DNA markers, in particular in HLA haplotypes enriched in Finland.

**Conclusions:**

The present study shows that HLA imputation in a blood donor biobank of reasonable size can be used to identify HLA homozygous blood donors suitable for cell therapy, HLA-typed thrombocytes and research. The homozygotes were HLA-compatible with a large fraction of the Finnish population. Regular blood donors reported to have positive attitude to research donation appear a good option for these purposes. Differences in population frequencies of HLA haplotypes emphasize the need for population-specific collections of HLA homozygous samples.

**Supplementary Information:**

The online version contains supplementary material available at 10.1186/s13287-022-03182-7.

## Background

Allogeneic therapeutic cells, such as induced pluripotent stem (iPS)-derived cell lines, mesenchymal stromal cells or immune cells [1, 2] may be readily rejected if they express human leucocyte antigen (HLA) alleles not found in the recipient [3–5]. Whole genome studies on hematopoietic stem cell transplantation indicate that full matching of the HLA alleles is the single, most important factor for cell compatibility between donor and recipient [6]; the same can be assumed for other cell therapies, albeit no comprehensive studies have been done so far. Searching cell donors with a full HLA identity to a recipient is demanding and requires vast donor pools, as demonstrated by international stem cell donor registries and their network [7]. Use of HLA homozygous cells has been suggested as an alternative at least in cases where cell lines could be used [8]. Cells from individuals who are homozygous for all major HLA genes, HLA-A, -B, -C, -DRB1, -DQA1, and -DQB1 can be assumed to be well tolerated by those who carry at least one copy of the HLA haplotype. A relatively small number of HLA homozygous donors has been shown to be compatible with a surprisingly large fraction of the population, making the approach feasible for e.g. iPS cell production [8–14].

As HLA genes are genetically extremely polymorphic [15], with thousands of alleles in each gene, finding individuals who are homozygous for all HLA genes requires large donor pools. Stem cell registries set up for clinical need for HLA identical stem cell donors for unrelated hematopoietic stem cell transplantation is one obvious source for donors. An alternative could be biobanks containing large numbers of genotyped blood donors. As determination of alleles of all HLA genes from a large number of blood donors is expensive, their HLA variation can alternatively be screened efficiently by using HLA imputation tools on genome data [16–23]. Once the potential homozygous donors have been identified, homozygosity can be confirmed by a clinical grade HLA typing method. Individuals regularly and voluntarily donating blood can be readily contacted by blood banks for an informed consent and fresh blood samples [24]. In addition, blood donors are known to have a positive attitude towards research [25, 26].

A homozygous donor pool or stored cell repository may be useful not only for iPS production but also for other cell therapies utilizing e.g. allogeneic mesenchymal stromal cells or immune cells. Many current immune cell therapies, particularly chimeric antigen receptor T cells, rely on autologous cells but in the future also allogeneic cells may be an option. HLA homozygous blood donors are also valuable for HLA-typed thrombocyte units that are administered for patients, such as leukaemia patients, who need large amounts of thrombocytes [27]. Matching of HLA-A and -B significantly lowers the risk of anti-HLA response and rejection. HLA homozygous cells are also valuable for immunogenetic studies and HLA peptide-binding studies [28, 29].

The present study utilizes a Finnish blood donor biobank resource with genome-level DNA marker data and methods [16, 18–23] set up for reliable HLA allele imputation [17]. Based on the genome data we imputed the HLA alleles of over 20,000 blood donors and identified individuals homozygous for all the seven classical HLA genes. These homozygotes included not only the typical European haplotypes but many HLA haplotypes reported to be enriched in Finland but rare elsewhere [30]. Hence, by this approach we were able to identify blood donors who are good candidates for acting as cell donors for research and future therapies.

## Methods

### Biobank genome data

Access to genotypes of 20,737 blood donors were granted by the Blood Service Biobank of the Finnish Red Cross Blood Service (FRCBS), Helsinki, Finland, decision 002–2018. Use of the samples and genotype data is in accordance with the biobank consent and meets the requirements of the Finnish Biobank Act 688/2012. The biobank samples, i.e. leftover samples of the diversion pouch, were collected along the standard blood donation from blood donors who had given a written biobank consent for the Blood Service Biobank.

The biobank samples were genotyped using the FinnGen ThermoFisher Axiom custom array v2 originally as part of the FinnGen project [31], that returned the genotypes back to the biobank (data release 4). Genotyping, quality control, and genome imputation protocols are described in detail in FinnGen GitBook [31]. In brief, genotype calling was performed with AxiomGT1 algorithm. Prior the imputation, genotyped samples were pre-phased with Eagle 2.3.5 with the default parameters, except the number of conditioning haplotypes was set to 20,000. Genotype imputation was performed using the population-specific imputation reference panel SISu v3 including 3,775 high coverage (25–30x) whole genome sequence data, with Beagle 4.1 (version 08Jun17.d8b).

### HLA imputation

HLA-A, -B, -C, -DRB1, -DQA1, -DQB1, -DPB1 alleles were imputed at high-resolution level using HIBAG algorithm [16] with population-specific models in genome build 38 as described by Ritari et al. [17]. The frequencies of imputed HLA alleles were calculated by using an annotated Rscript by Mark Christie [32]. Correlation between imputed allele frequencies and reference allele frequencies were measured with Pearson’s product–moment correlation.

Posterior probabilities (PP) of the imputed HLA alleles in each identified haplotype are shown in Additional file 1: Fig. 1. Altogether 27,588 clinical grade HLA typing results at high resolution level (unpublished data, Stem Cell Registry, Finland) were available from 13,794 individuals of the present cohort. The number of errors varied within a range of 0–4.6% (Additional file 2: Table 2). One sample identified as homozygous by imputation for the seven classical HLA genes, was found to be heterozygous in HLA-C locus in this comparison analysis and was therefore excluded from the further analyses. Correlation of the allele frequencies of the imputed alleles in the present biobank cohort and allele frequencies of the Stem Cell Registry Finland (unpublished data) is shown in Additional file 3: Fig. 3. There was no reference frequency for the alleles HLA-C*17:03 and HLA-DPB1*105:01; therefore, these two alleles, as well as the alleles of which imputation result remained in low resolution level, were excluded from the correlation analyses (Additional file 4: Table 4).

**Fig. 1:**
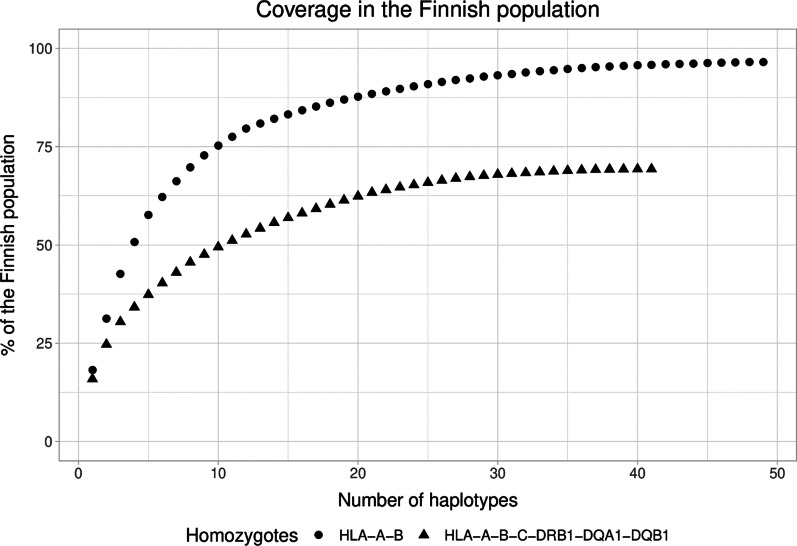
Percentages of the Finnish population estimated to be HLA-compatible with HLA-A-B homozygous and HLA-A to DQB1 homozygous cell donors

Additional verification of the homozygosity of the imputed HLA haplotypes was performed by clinical HLA typing of 30 samples (Table 1) in the EFI-accredited HLA laboratory of the Finnish Red Cross Blood Service, Helsinki, Finland. HLA typing was performed using the targeted PCR based on NGS technique according to the protocol provided by the manufacturer (NGSgo Workflow, GenDx, Utrecht, The Netherlands). The allele assignment at the four-field resolution level was implemented by NGSengine version 2.11.0.11444 (GenDx, Utrecht, The Netherlands) using IPD IMGT/HLA database, release 3.33.0; [33].

**Table 1:** Homozygous HLA-A-B-C-DRB1-DQA-DQB1 haplotypes, level of homozygosity within a haplotype and number of blood donors identified among 20,737 blood donors

Haplotype	HLA-A	HLA-B	HLA-C	HLA-DRB1	HLA-DQA1	HLA-DQB1	N	Observed frequency	Expected frequency	Heterozy-gous markers %
1	03:01	35:01	04:01	01:01	01:01	05:01	149	0.00718522	0.00690815	0.12
2	01:01	08:01	07:01	03:01	05:01	02:01	48	0.00231470	0.00242061	0.41
3	03:01	07:02	07:02	15:01	01:02	06:02	27	0.00130202	0.00113331	0.64
4*	02:01	07:02	07:02	15:01	01:02	06:02	7	0.00033756	0.00051551	2.11
5*	02:01	13:02	06:02	07:01	02:01	02:02	7	0.00033756	0.00039721	0.67
6*	02:01	15:01	03:04	04:01	03:01	03:02	7	0.00033756	0.00029789	1.23
7	03:01	07:02	07:02	13:01	01:03	06:03	6	0.00028934	0.00035215	1.62
8	02:01	27:05	02:02	08:01	04:01	04:02	5	0.00024111	0.00031656	0.01
9* F	02:01	51:01	15:02	09:01	03:02	03:03	5	0.00024111	0.00013848	0.02
10 F	03:01	15:01	03:03	08:01	04:01	04:02	5	0.00024111	0.00007897	0.02
11	02:01	15:01	03:03	13:01	01:03	06:03	4	0.00019289	0.00017316	2.31
12	24:02	40:01	03:04	13:02	01:02	06:04	4	0.00019289	0.00011964	0.12
13	02:01	27:05	01:02	01:01	01:01	05:01	4	0.00019289	0.00011769	1.16
14	02:01	15:01	04:01	08:01	04:01	04:02	3	0.00014467	0.00018545	0.09
15* F	03:01	18:01	07:01	04:04	03:01	03:02	3	0.00014467	0.00007943	0.02
16	02:01	08:01	07:01	03:01	05:01	02:01	3	0.00014467	0.00006399	2.21
17 F	31:01	18:01	07:01	15:01	01:02	06:02	2	0.00009645	0.00013433	0.03
18 F	03:01	07:02	03:04	01:01	01:01	05:01	2	0.00009645	0.00008789	2.43
19	02:01	40:01	03:04	13:02	01:02	06:04	2	0.00009645	0.00007268	2.35
20 F	24:02	39:01	07:02	08:01	04:01	04:02	2	0.00009645	0.00002717	2.90
21 F	02:01	15:01	04:01	15:01	01:02	06:02	2	0.00009645	0.00000850	0.40
22	02:01	44:02	05:01	12:01	05:05	03:01	1	0.00004822	0.00007523	5.20
23	68:01	08:01	07:01	03:01	05:01	02:01	1	0.00004822	0.00006170	0.003
24 F	02:01	27:05	01:02	04:08	03:03	03:01	1	0.00004822	0.00003359	0.03
25	24:02	35:01	04:01	01:01	01:01	05:01	1	0.00004822	0.00002598	0.02
26 F	02:01	56:01	01:02	04:01	03:01	03:02	1	0.00004822	0.00002582	0.01
27	03:01	15:01	03:04	04:01	03:01	03:02	1	0.00004822	0.00002199	0.06
28* F	31:01	51:01	01:02	13:01	03:02	03:03	1	0.00004822	0.00002156	0.40
29 F	02:01	56:01	01:02	15:01	01:02	06:02	1	0.00004822	0.00001116	0.03
30	02:01	27:05	02:02	01:01	01:01	05:01	1	0.00004822	0.00000611	6.73
31	29:02	44:03	16:01	07:01	02:01	02:02	1	0.00004822	0.00000374	0.08
32	23:01	44:03	04:01	07:01	02:01	02:02	1	0.00004822	0.00000318	0.25
33	32:01	40:02	03:04	14:02	05:03	03:01	1	0.00004822	0.00000300	0.14
34	03:01	15:01	03:03	13:02	01:02	06:04	1	0.00004822	0.00000290	0.01
35	32:01	35:01	04:01	04:01	03	03:01	1	0.00004822	0.00000218	0.07
36	11:01	44:02	05:01	04:04	03:01	03:02	1	0.00004822	0.00000146	0.03
37	32:01	44:02	05:01	15:01	01:02	06:02	1	0.00004822	0.00000127	0.06
38	11:01	35:01	03:03	08:01	04:01	04:02	1	0.00004822	0.00000048	0.06
39	25:01	08:01	07:01	03:01	05:01	02:01	1	0.00004822	0.00000008	0.08
40	26:01	40:02	03:04	08:01	04:01	04:02	1	0.00004822	0.00000004	0.12
41	02:01	51:01	15:02	04:01	03:01	03:02	1	0.00004822	0.00000001	0.03

*Haplotypes are listed according to the number of homozygous individuals identified. N *Number of individuals homozygous for the haplotype. The expected frequency refers to the calculated frequency (haplotype frequency *p*^2^) of each homozygote based on haplotype frequencies of the Finnish Stem Cell Registry. Heterozygous markers % refers to the average percentage of heterozygous markers found within a given haplotype. * = homozygosity of all the samples representing the haplotype confirmed by clinical grade HLA typing. FER = haplotype enriched in the Finnish population but rare elsewhere as described in Linjama et al. [30]

### Haplotype coverage

Haplotype coverage in the Finnish population was calculated based on the formulas published by Gourraud et al*.* 2012 [14]. Based on known haplotype frequencies in Finland ([34], unpublished data, Stem Cell Registry, Finland) we calculated the percentage of the Finnish population compatible with identified homozygotes. We first calculated the proportion of the Finnish population carrying each identified HLA haplotype. We then calculated the overlap portion, in other words, what part of the population would be compatible with more than one haplotype. By taking into account both, the proportion of the Finnish population carrying the identified HLA haplotype and the overlap of the population that would be compatible with multiple of identified HLA haplotypes, we were able to calculate what percentage of the Finnish population would be compatible with at least one of the identified HLA haplotypes. The population coverage was calculated for HLA-A-B (low resolution, 2 digit) and HLA-A-B-C-DRB1-DQ (high resolution, 4 digit) identified haplotypes.

### MHC-located DNA polymorphism markers

Homozygosity levels of 36,161 DNA polymorphism markers located on the major histocompatibility complex (MHC) chromosomal segment encompassing human genome segment (hg38): 29,942,470–32,666,689, i.e. the HLA-A-to-DQB1 segment, was determined for all the 317 individuals found homozygous for HLA-A, -B, -C, -DRB1, -DQA1, and -DQB1 genes. We further determined the level of homozygosity in 60,808 DNA markers located in the extended MHC segment (hg38): 28,510,120–33,480,577, in individuals homozygous from HLA-A to HLA-DPB1 in ten most common haplotypes (Table 2). Percentages of the heterozygous markers in these segments were calculated.

**Table 2:** 10 most common homozygous HLA-A-B-C-DRB1-DQA1-DQB1-DPB1 haplotypes, level of homozygosity in the MHC region by haplotype and number of blood donors identified among 20,737 blood donors

Haplotype	HLA-A	HLA-B	HLA-C	HLA-DRB1	HLA-DQA1	HLA-DQB1	HLA-DPB1	*N*	Observed frequency	Expected frequency	Heterozygous markers %
1	03:01	35:01	04:01	01:01	01:01	05:01	04:02	71	0.00342	0.00309	0.31
2	01:01	08:01	07:01	03:01	05:01	02:01	01:01	11	0.00053	0.00061	1.70
3	03:01	07:02	07:02	15:01	01:02	06:02	04:01	14	0.00068	0.00058	1.99
4*	02:01	07:02	07:02	15:01	01:02	06:02	04:01	2	0.00010	0.00005	5.85
5*	02:01	13:02	06:02	07:01	02:01	02:02	04:01	7	0.00034	0.00031	1.15
6*	02:01	15:01	03:04	04:01	03:01	03:02	04:01	4	0.00019	0.00024	3.43
7	03:01	07:02	07:02	13:01	01:03	06:03	04:01	2	0.00010	0.00011	0.05
8	02:01	27:05	02:02	08:01	04:01	04:02	03:01	4	0.00019	0.00025	0.82
9* FER	02:01	51:01	15:02	09:01	03:02	03:03	04:02	4	0.00019	0.00012	0.13
10 FER	03:01	15:01	03:03	08:01	04:01	04:02	03:01	4	0.00019	0.00004	0.47

The haplotypes are listed in the same order as in Table 1. The most common homozygous HLA-DPB1 allele in 10 most common HLA-A-B-C-DRB1-DQA1-DQB1-DPB1 homozygotes identified among 20,737 blood donors was taken into consideration. *N *Number of individuals homozygous for the haplotype. The expected frequency refers to the calculated frequency (haplotype frequency p^2^) of each homozygote based on haplotype frequencies of Linjama et al. [37]. Heterozygous markers % refers to the average percentage of heterozygous markers found in the MHC region by haplotype. * = homozygosity of all the samples representing the haplotype confirmed by clinical grade HLA typing. FER = haplotype enriched in the Finnish population but rare elsewhere as described in Linjama et al. [30]

### Code availability

All analyses in this study were performed with R version 3.6.1 [35] with RStudio [36]. Analysis scripts are available at https://github.com/FRCBS/HLA_homozygosity.

## Results

### Homozygotes for HLA-A, -B, -C, -DRB1, -DQA and -DQB1

Altogether 317 of 20,737 (1.5%) blood donors were identified as homozygous for imputed HLA-A, -B, -C, -DRB1, -DQA1 and -DQB1 alleles (Table 1). Forty-one different HLA haplotypes were observed, and of these 21 were found in more than one individual. No HLA haplotypes that haven’t been met in Finland before were identified. The most common HLA-A to -DQB1 homozygotes, A3 ~ B35 ~ DR1 ~ DQ5 (*n* = 149 homozygotes), A1 ~ B8 ~ DR3 ~ DQ2 (*n* = 48) and A3 ~ B07 ~ DR15 ~ DQ6 (*n* = 27), were those known to be common in many European populations. Eleven homozygotes for HLA haplotypes enriched in Finland but very rare elsewhere were found (marked by FER in Table 1). The observed frequencies of homozygotes among the present cohort of blood donors did not differ from those estimated from the haplotype frequencies of the Finnish Stem Cell Donor Register (Table 1).

We confirmed the HLA imputation results by typing the seven classical HLA alleles in 30 homozygous samples (Table 1) representing six different HLA-A-to-DQ haplotypes by clinical grade method. All the clinical typing results matched 100% to the imputed result. Posterior probabilities (PP) of the imputed HLA alleles in each haplotype are shown in Additional file 1: Fig. 1. They all remained over the cut off value of 0.5, except for HLA-A 02:01 in haplotype 11 (lowest PP = 0.495) and HLA-DRB1 08:01 in haplotype 40 (PP = 0.492).

We next analysed homozygosity levels of 36,161 MHC-located polymorphic DNA markers in the 317 HLA homozygotes to reveal possible non-HLA genomic variation in the MHC segment. For the 10 most common HLA haplotypes, i.e. those with at least 5 individuals, the average levels of heterozygous markers in the genome segment (hg38): 29,942,470–32,666,689 were between 0.01% (haplotype 8 in Table 1) to 2.11% (haplotype). There was no clear correlation between heterozygosity level and frequencies of the haplotypes. However, haplotypes 9 and 10 that belong to the Finnish enriched haplotypes had low heterozygosity levels, 0.02% both.

### Homozygotes for HLA-A, -B, -C, -DRB1, -DQA, -DQB1 and -DPB1

We extended the homozygosity analysis to the DPB1 gene, located centromeric to the DQB1 gene. Both the number of different HLA-A-to-DPB1 identified haplotypes (*n* = 38), and the number of individuals (*n* = 160) were lower than those for HLA-A-to-DQ homozygotes (41 and 317, respectively). The most common DPB1 allele found as homozygous in the 10 most frequent HLA-A-to-DQ haplotypes is shown in Table 2.

The extended, HLA-DPB1 locus containing MHC segment (hg38: 28,510,120–3,480,577) contained 60,808 DNA markers. The level of homozygosity in HLA-A-to-DPB1 homozygotes was analysed in the ten most common HLA-A-DPB1 haplotypes (Table 2). In general, the average levels of heterozygosity remained at the same levels, or perhaps with a tendency for some increase, as among the HLA-A-to-DQ homozygotes (Table 1).

### Homozygotes for HLA-A and -B

Of the 20,737 blood donors, 4156 were homozygous for imputed HLA-A and 1998 for HLA-B at low resolution level generally used for HLA-matched thrombocytes. Altogether 741 of 20,737 (3.6%) individuals were identified homozygous for both HLA-A and HLA-B at low resolution level. Forty-nine different low-resolution HLA-A-B haplotypes were observed as homozygous (Table 3).

**Table 3:** Homozygous low-resolution HLA-A and -B haplotypes and number of blood donors found among 20,737 blood donors based on HLA imputation

Haplotype	HLA-A	HLA-B	*N*	Observed frequency	Expected frequency
1	03	35	195	0.00940	0.009106
2	03	07	135	0.00651	0.005687
3	02	15	107	0.00516	0.005127
4	01	08	62	0.00299	0.003129
5	02	27	54	0.00260	0.002584
6	02	44	26	0.00125	0.000954
7	02	07	22	0.00106	0.001288
8	02	40	22	0.00106	0.001146
9	03	15	22	0.00106	0.000803
10	02	51	12	0.00058	0.000507
11	24	40	11	0.00053	0.000526
12	02	13	7	0.00034	0.000595
13	24	39	6	0.00029	0.000217
14	02	56	5	0.00024	0.000180
15	24	07	5	0.00024	0.000161
16	31	18	3	0.00014	0.000166
17	03	18	3	0.00014	0.000149
18	02	08	3	0.00014	0.000097
19	11	35	2	0.00010	0.000112
20	11	44	2	0.00010	0.000091
21	68	51	2	0.00010	0.000058
22	31	51	2	0.00010	0.000043
23	32	40	2	0.00010	0.000043
24	68	35	2	0.00010	0.000036
25	68	44	2	0.00010	0.000038
26	26	40	2	0.00010	0.000021
27	69	08	2	0.00010	0.000082
28	02	35	1	0.00005	0.000191
29	24	15	1	0.00005	0.000122
30	02	18	1	0.00005	0.000107
31	24	35	1	0.00005	0.000104
32	25	18	1	0.00005	0.000081
33	03	27	1	0.00005	0.000086
34	03	44	1	0.00005	0.000056
35	32	44	1	0.00005	0.000044
36	03	40	1	0.00005	0.000047
37	03	51	1	0.00005	0.000042
38	31	40	1	0.00005	0.000020
39	31	39	1	0.00005	0.000014
40	11	55	1	0.00005	0.000008
41	01	07	1	0.00005	0.000009
42	29	44	1	0.00005	0.000007
43	32	35	1	0.00005	0.000007
44	23	44	1	0.00005	0.000007
45	01	39	1	0.00005	0.000004
46	01	51	1	0.00005	0.000006
47	24	51	1	0.00005	0.000007
48	68	15	1	0.00005	0.000005
49	24	08	1	0.00005	0.000002

The haplotypes are listed according to the number of homozygous individuals identified. *N* = number of individuals homozygous for the haplotype. The expected frequency refers to the calculated frequency of each homozygote (haplotype frequency *p*^2^) based on the haplotype frequencies of the Finnish Stem Cell Registry

### Identified haplotype coverage in Finland

Percentages of the Finnish population compatible with the cells from identified HLA-A-to-DQ and HLA-A-B homozygotes were calculated based on the haplotype frequencies of the Finnish Stem Cell Donor Register (unpublished data, Stem Cell Registry, Finland).

Ten most frequent HLA-A-to-DQB1 homozygotes were estimated to be HLA-compatible with 49.5% of the Finnish population (Fig. 1) and the three most common ones, found as homozygous in 224 individuals of the present cohort, were HLA-compatible with 30.4% of the population. The overall cumulative coverage of the 41 different HLA-A-to-DQB1 homozygotes found in the present study of 20,737 blood donors was 69.3% of the Finnish population.

Ten most frequent HLA-A-B homozygotes were HLA low resolution compatible with 75.3% of the Finnish population (Fig. 1) and the three most common ones, found as homozygous in 437 individuals, to 42.6% of the population. The overall cumulative coverage of the 49 different HLA-A-B homozygotes identified in the present study of 20,737 blood donors was 96.6% of the Finnish population.

## Discussion

The present study shows that a blood donor biobank genome data in combination with HLA imputation is a powerful resource for the identification of individuals homozygous for HLA alleles. As fresh blood samples may be needed for further studies utilizing HLA homozygosity, blood donors are a very valuable population as they voluntarily and regularly donate blood. Blood establishments usually have efficient high-quality pipelines for collecting and handling the samples. Furthermore, as blood donors have a positive attitude to research [25, 26], fresh samples can be requested more readily than e.g. from patients.

HLA homozygous blood cells can be utilized in several ways. Firstly, as suggested by Taylor et al. [8] and Nakatsuji et al. [9] and others, the HLA homozygotes are a good source for HLA-compatible embryonic stem cells and iPS cells. Several estimates of population coverage at various HLA matching levels in diverse populations have been reported [8, 10–14]. It has been estimated, that approximately 20,000 HLA homozygotes would be needed to reach ~ 50% coverage in European populations [14]. In Spain, the population coverage estimations of HLA-A, -B and -DRB1 homozygous donors for 41 haplolines have been shown to be 50% of the Spanish population, and 190 homozygous haplolines for HLA-A, -B, -C, -DRB1 and -DQB1 was estimated to cover 70% of the Spanish population [13]. The level of population coverage in our study was very high; 41 donors homozygous for HLA-A, -B, -C, -DRB1, -DQA1 and -DQB1 was enough to reach a cumulative coverage of 69.3% in the Finnish population. Furthermore, to our knowledge, no analysis including high-resolution allele data of all six major HLA genes HLA-A, -B, -C, -DRB1, -DQA and -DQB1 has been performed. Extending the matching criteria to HLA-DPB1 may be challenging due to the recombination hot spot between HLA-DQB1 and HLA-DPB1 [15]. In the present study, HLA-A to HLA-DPB1 haplotypes and haplotype frequencies (Table 2) were equivalent to the HLA-A-DPB1 haplotypes identified earlier in the Finnish population [37].

Secondly, the HLA-A and -B homozygotes are a good source for HLA-matched thrombocytes. HLA matching is not considered in standard platelet transfusion, but for patients in need of high amounts of platelets HLA-A and -B mismatching is considered a major cause for alloimmunization due to expression of HLA class I molecules on platelets and consequent poor response. Furthermore, anti-HLA antibodies may result in platelet refractoriness in later platelet transfusions [38, 39]. The overall cumulative coverage of the 49 different HLA-A-B homozygotes identified in the present study of 20,737 blood donors was as high as 96.6% of the Finnish population, indicating that a surprising low number of selected blood donors in theory were needed. However, the other aspects and requirements of thrombopheresis must be considered for real-life donor pool; hence, much higher numbers of blood donors are needed. We are currently evaluating how the HLA imputation could be used in actual pool of thrombocyte donors. In addition, the type of SNP array developed by e.g. the Blood transfusion Genomics Consortium [40] may in the future produce the low-resolution HLA types from all blood donors if used for general blood alloantigen typing platform.

Thirdly, the HLA homozygote cells may be a good option for various allogeneic immune cell therapies if avoidance of immunological rejection is warranted. In all these three examples, the key advantage is that HLA homozygous cells are HLA-compatible with all those who carry at least one copy of the homozygous HLA alleles. As shown in the present study and in other earlier reports, a surprisingly low number of donors is needed for a good population coverage. The actual number of course depends on the genetic structure of the population and on the level of required HLA matching. For HLA-typed thrombocytes low-resolution HLA-A and -B matching is needed, whereas for stem cell therapies more stringent matching is needed. Despite of the good posterior probability values in this study, confirmatory clinical grade HLA typing is needed prior any therapeutic use.

Finally, availability of HLA homozygous blood cells via a biobank is valuable for immunological and immunogenetic research. For example, to study peptide repertoire presented by each HLA allele benefits from HLA homozygous cell samples [29]. The ten most common identified HLA haplotypes of the present study had e.g. seven different HLA-B and eight different DRB1 alleles, thus providing a good selection of alleles for research purposes.

The HLA homozygosity may encompass the entire MHC segment, as demonstrated in the present study: the HLA homozygotes were highly homozygous for almost all > 60,000 MHC-located DNA markers. The HLA homozygous individuals, at least in Finland, can therefore be expected to be homozygous also for the many other genes of the MHC segment. Many of these genes are immunologically very interesting, for example, MICA and MICB as NK cell ligands, the complement component C4 genes, or HLA-G in immune suppression. These genes belong to gene families with highly similar gene sequences and show gene copy number variation. Genomic studies on HLA homozygotes may help and simplify these genome structure studies and can be used for functional analyses as well. Indeed, the International HLA Workshop is collecting HLA homozygous samples for genomic sequencing [41].

Small founder population, geographical and linguistic isolation and the population history of Finland have resulted in a distinct genetic structure of the Finnish population [42, 43]. Compared to other European populations, the Finns have shown to display significantly lower genetic diversity [44] and geographical genetic substructure of the population [45]. The genetic uniqueness of the Finns has previously been shown also in the HLA region by population-specific HLA allele and haplotype frequencies [34, 46] as well as by the enrichment of certain HLA haplotypes [30]. These so-called Finnish enriched rare haplotypes, FER haplotypes, are known to occur in Finland with much higher frequency than elsewhere. All FER haplotypes found as homozygous in the present study were at least 18 times more common in Finland than in Germany [30]. This fact strongly indicates that, albeit samples homozygous for the most common European haplotypes can be collected in many countries, population-specific sampling may be needed for a good population coverage. Two of the ten most common homozygotes in the present study are FER haplotypes that may not be readily found in other populations. It is of note that six of the identified HLA haplotypes found in the present study are among the ten most common HLA haplotypes in European populations [47]. These six HLA haplotypes were HLA-compatible with no less than 26.7% of Europeans. Therefore, therapeutic cell products derived from these donors would serve not only Finnish population, but in fact also a remarkable part of the European population.

## Conclusions

Allogeneic therapeutic cells are readily rejected if they express HLA alleles not found in the recipient. As finding cell donors with a full HLA match to a recipient requires vast donor pools, use of HLA homozygous cells has been suggested as an alternative. HLA homozygous cells should be well tolerated by those who carry at least one copy of donor HLA alleles. HLA-A-B homozygotes could be valuable for HLA-matched thrombocyte products. The present study shows that imputation of HLA alleles based on genome polymorphism data in a blood donor biobank of > 20,000 blood donors can be used to identify HLA homozygous blood donors suitable for cell therapy, HLA-typed thrombocytes and for many research purposes. The homozygotes were estimated to be HLA-compatible with a large fraction of the Finnish population. Regular blood donors who have been reported to have a positive attitude to research donation appear a good option for these purposes. Differences in population frequencies of HLA haplotypes emphasize the need for population-specific collections of HLA homozygous samples.

## Supplementary Information

**Additional file 1: Figure 1**Posterior probabilities of the imputed HLA alleles in a given HLA haplotype. Number of individuals homozygous for each haplotype (1-41) is stated in Table 1. Median, highest and lowest values of posterior probabilities are shown in haplotypes 1-21, and the actual posterior probability value in haplotypes (22-41) where one individual was identified.

**Additional file 2: Table 2.** Comparison of clinical grade and imputed HLA typing result. Altogether clinical grade result was available in 27,588 typings of 13,794 individuals of the present study. Only typing results where discrepancies occurred are shown in this table.

**Additional file 3: Figure 3.** Correlation analysis of the allele frequencies of the imputed data and the reference data.

**Additional file 4: Table 4.** Additional Table 4 HLA allele count and allele frequencies in imputed data set (N = 20737). Corresponding known HLA allele frequencies in Finland (Stem Cell Registry, unpublished data) are shown as reference allele frequencies. No reference allele frequency was available for the alleles HLA-C*17:03 and HLADPB1* 105:01 or for the alleles of which imputation result remained in low resolution level.

## Data Availability

The data that support the findings of this study are available from the Finnish Red Cross Blood Service Biobank, but restrictions apply to the availability of these data, which were used under licence for the current study, and so are not publicly available. Data are, however, available from the authors upon reasonable request and with permission of the Finnish Red Cross Blood Service Biobank.

## Abbreviations

MHC: Major histocompatibility complex
HLA: Human leucocyte antigen
iPS: Induced pluripotent stem cell
SNP: Single nucleotide polymorphism
PP value: Posterior probability value
