# Supplementary material for: Blood donor biobank and HLA imputation as a resource for HLA homozygous cells for therapeutic and research use
Source: Stem Cell Res Ther. 2022 Oct 9;13:502. doi: 10.1186/s13287-022-03182-7 (PMC9549658; doi:10.1186/s13287-022-03182-7)

**Additional Figure 1** Posterior probabilities of the imputed HLA alleles in a given HLA haplotype. Number of individuals homozygous for each haplotype (1-42) is stated in Table 1.

Median, highest and lowest value of posterior probabilities is shown in haplotypes 1-21, and the actual posterior probability value in haplotypes (22-42) where one individual was identified.


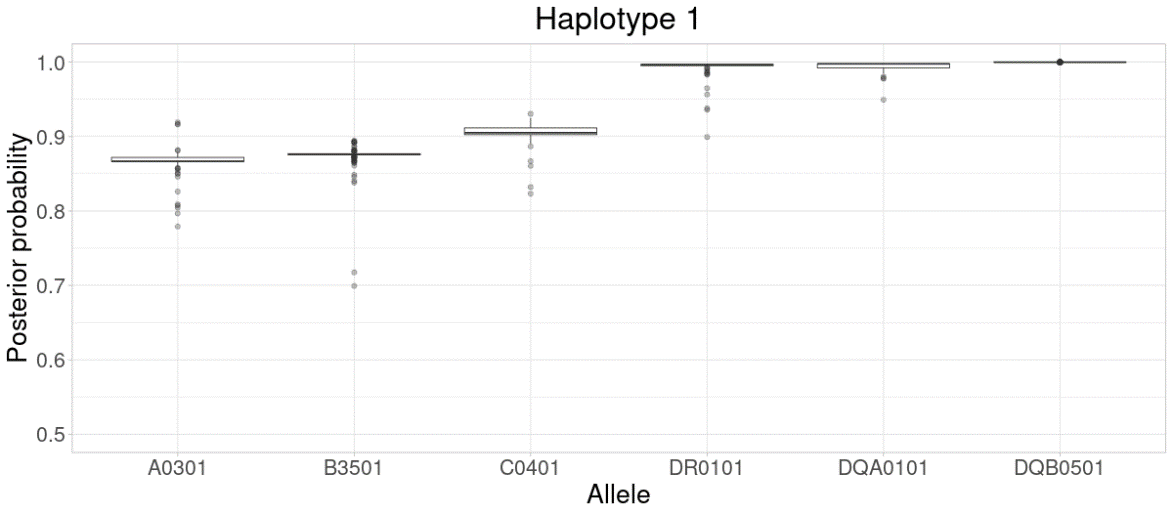

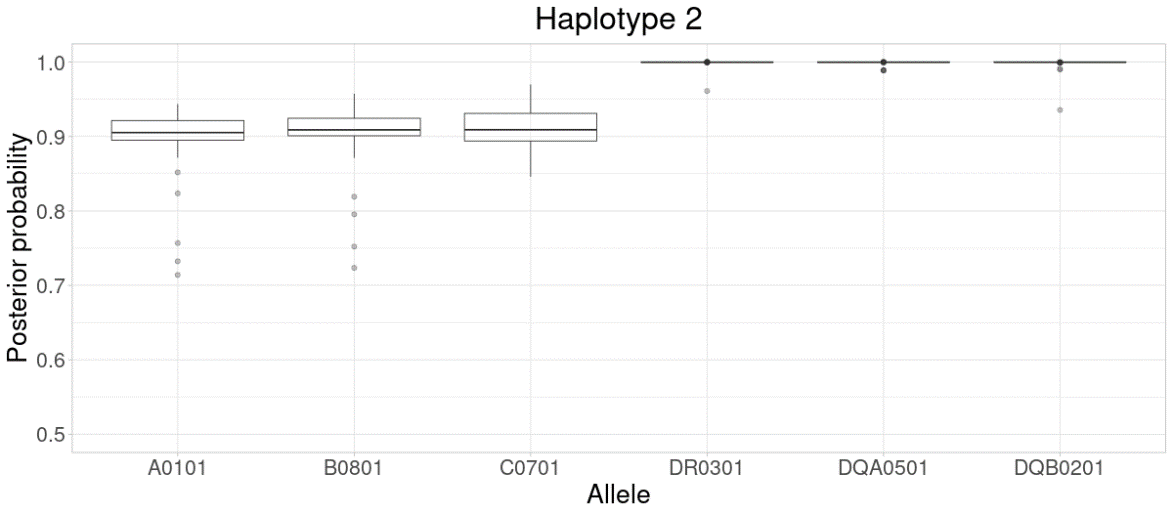


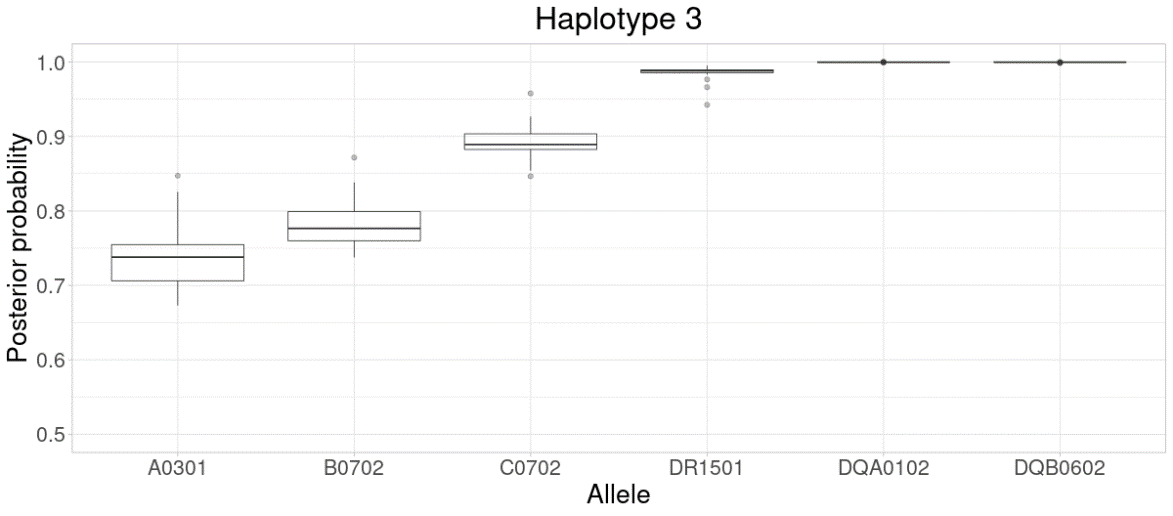

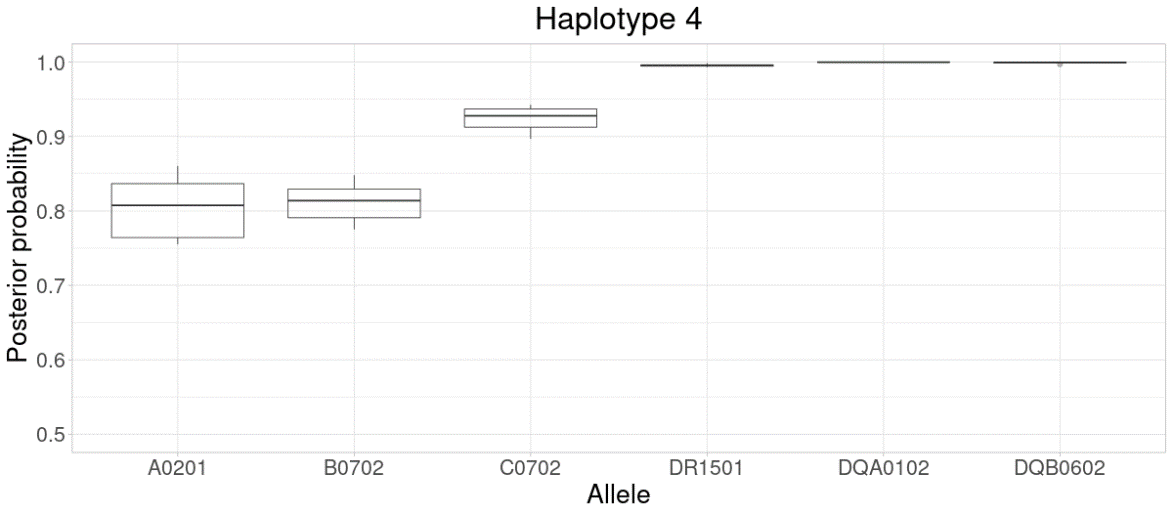


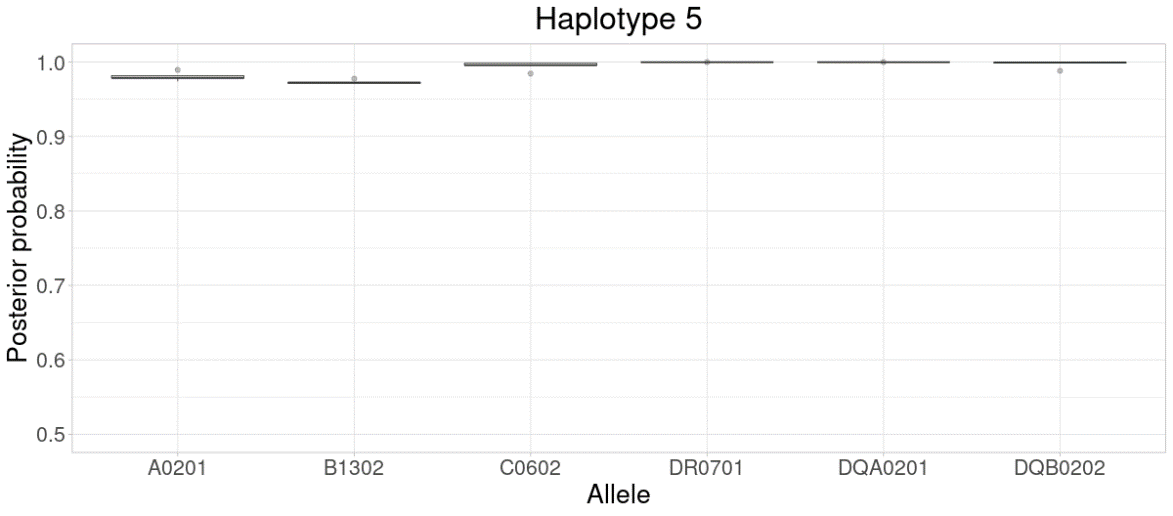

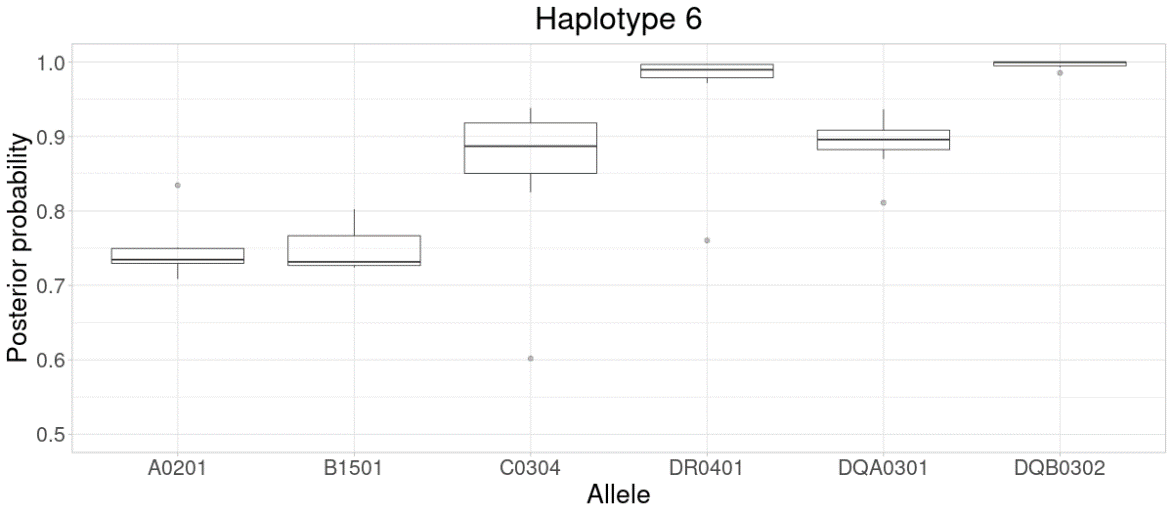


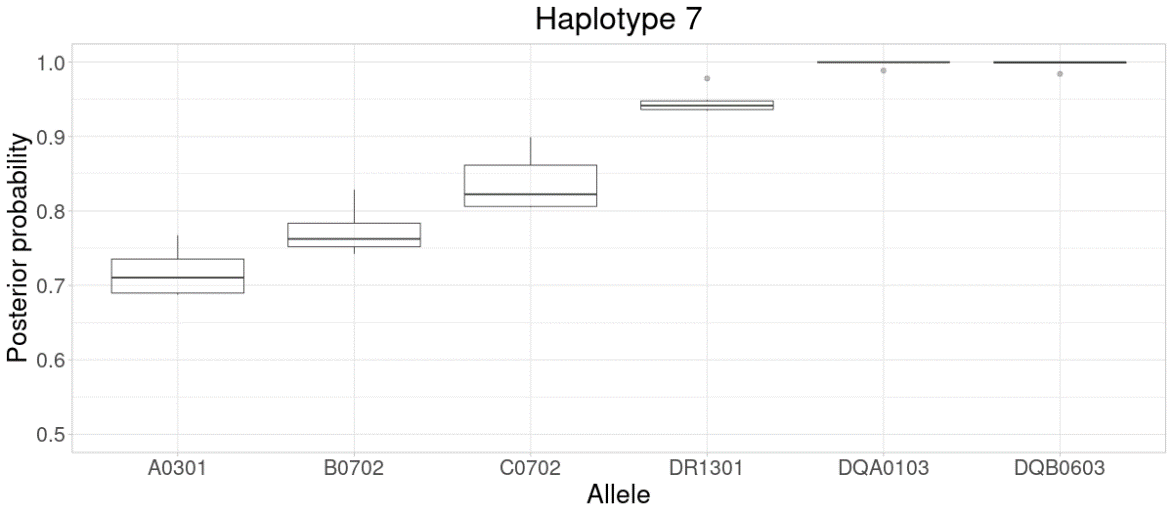

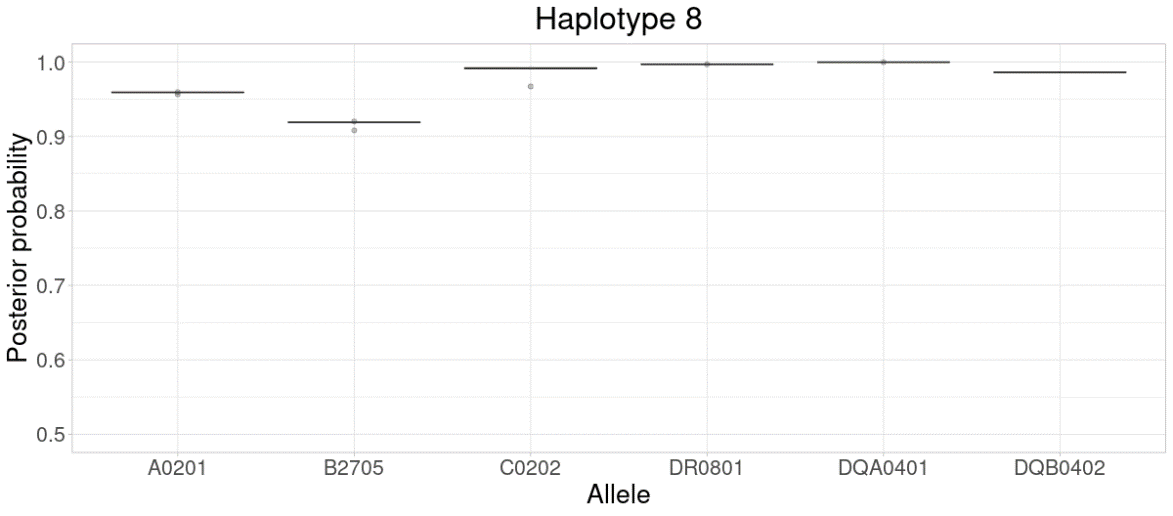


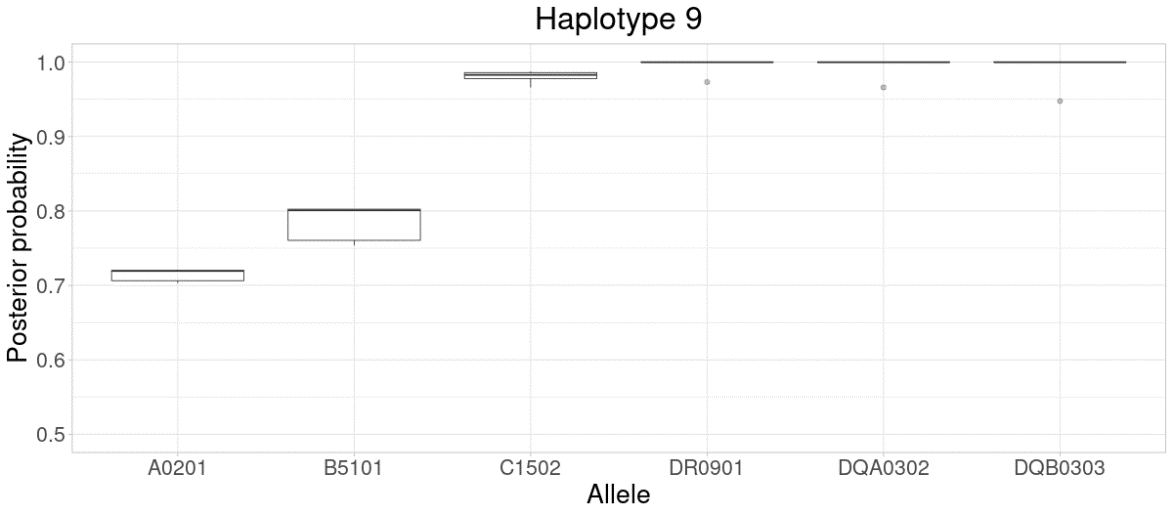

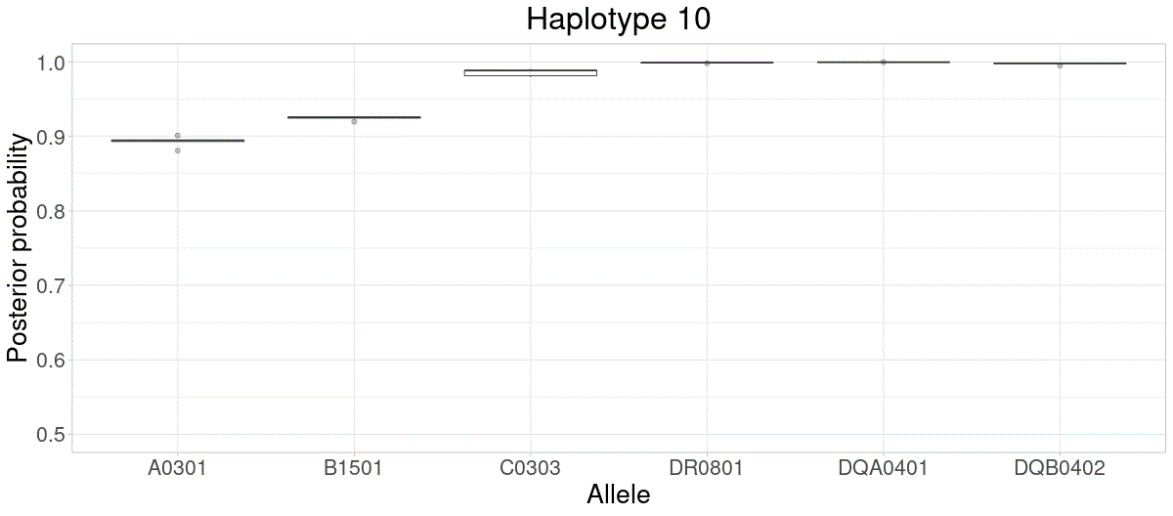


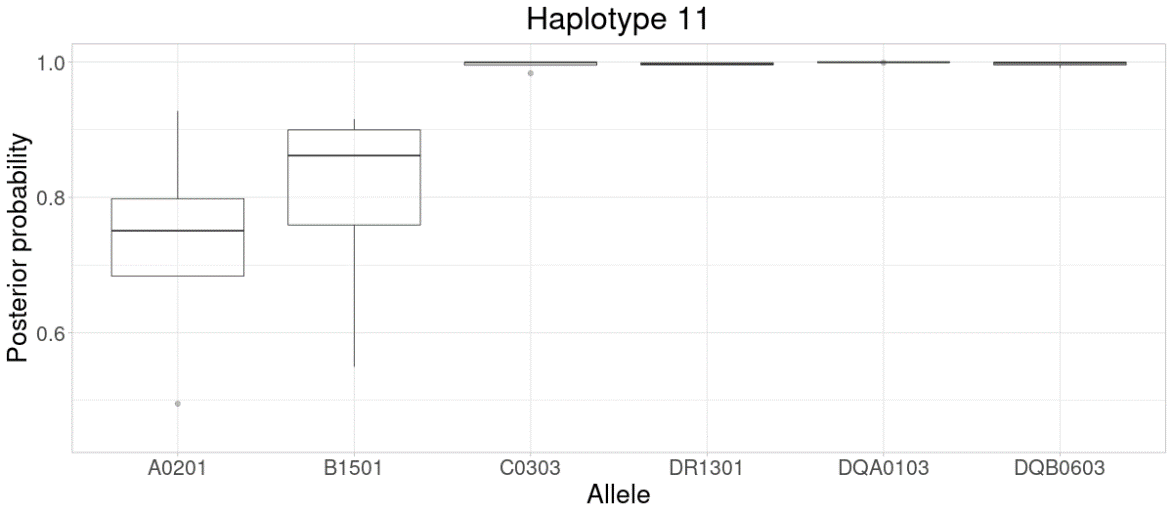

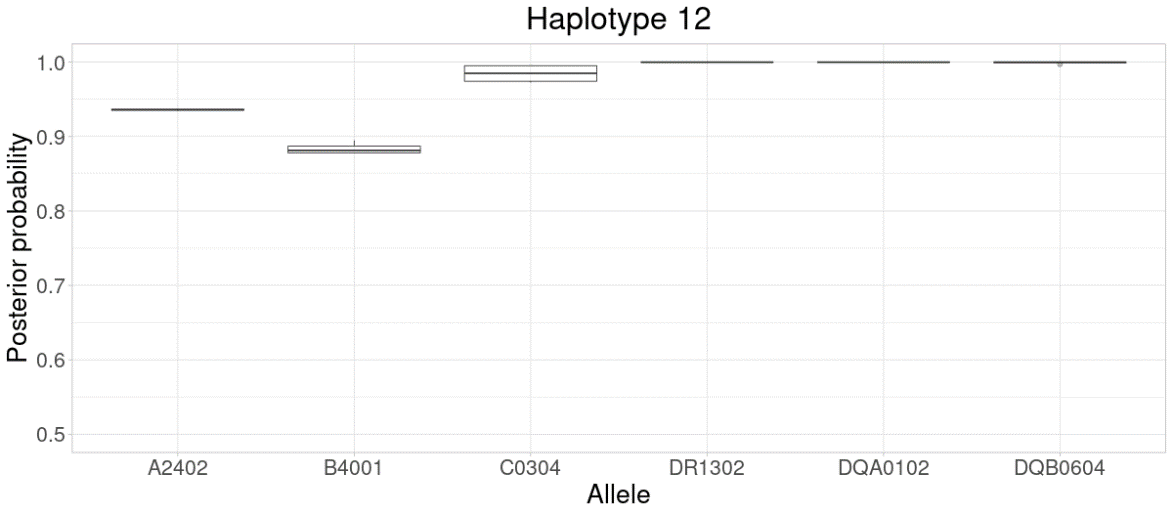


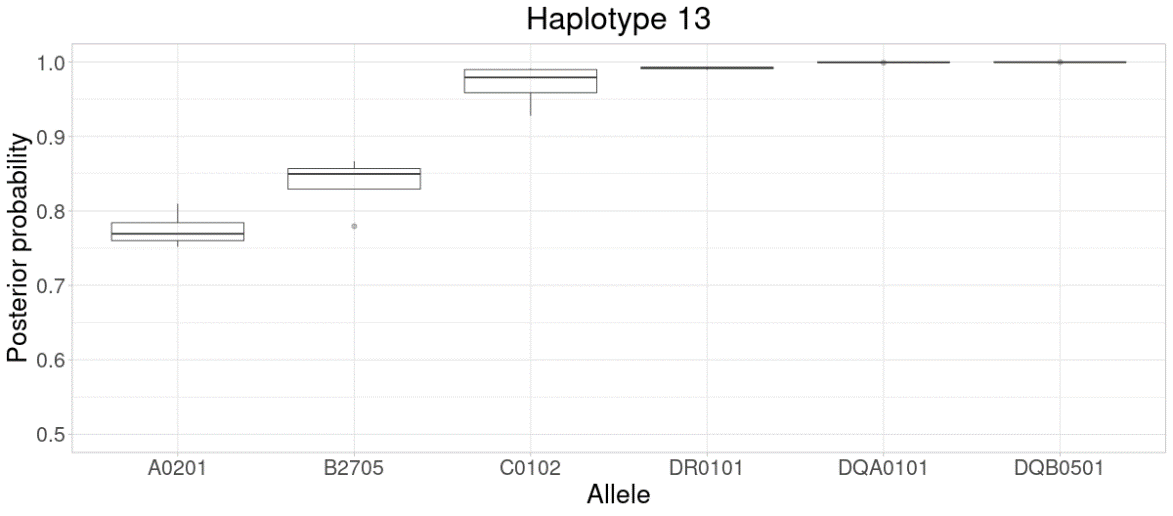

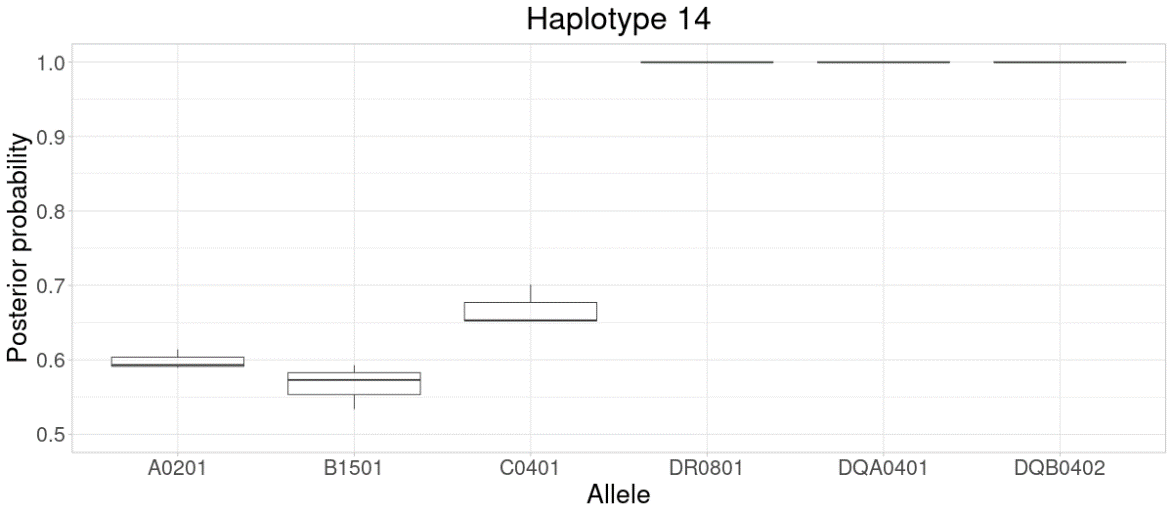


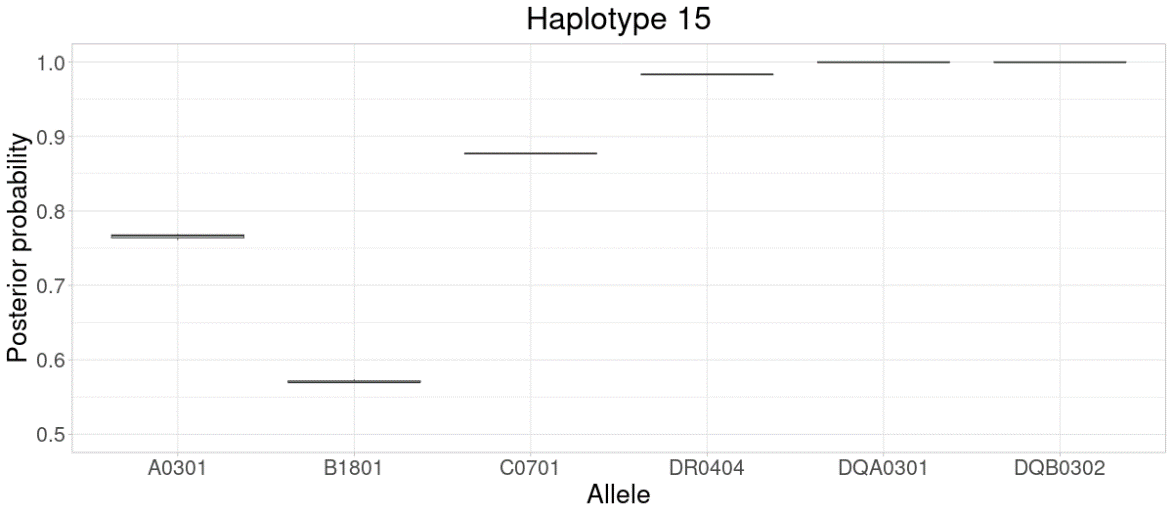

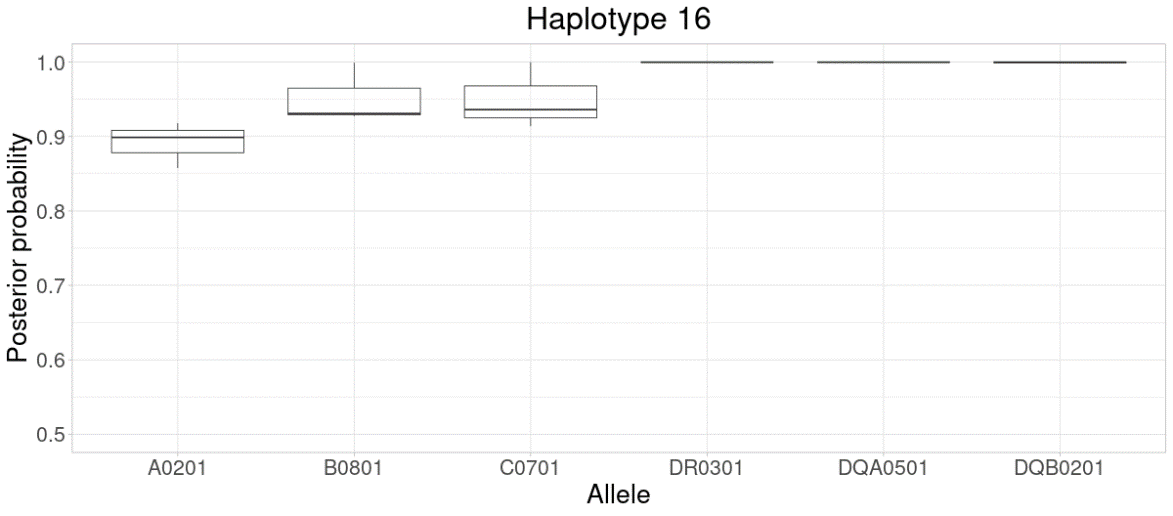


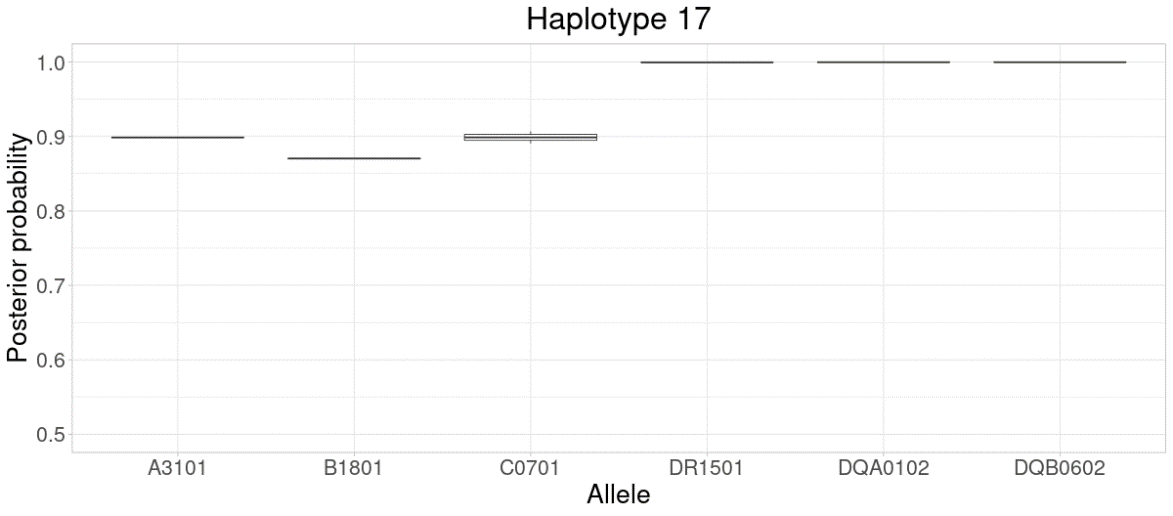

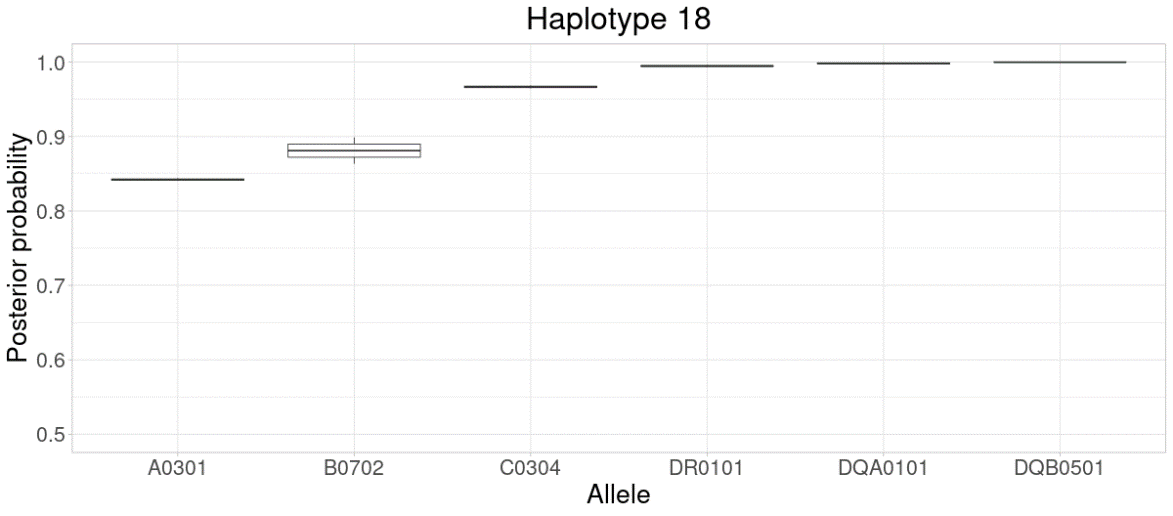


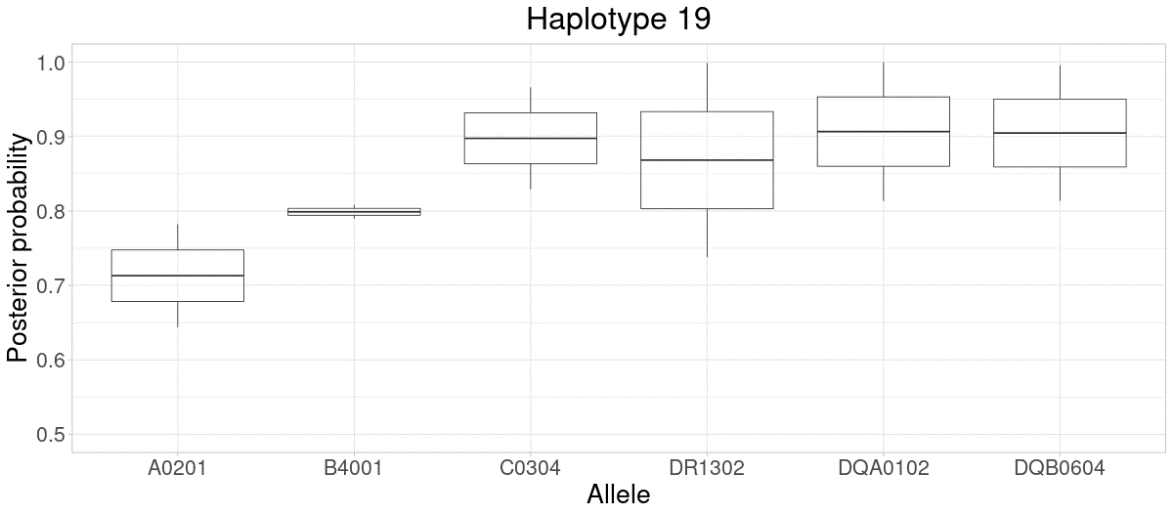

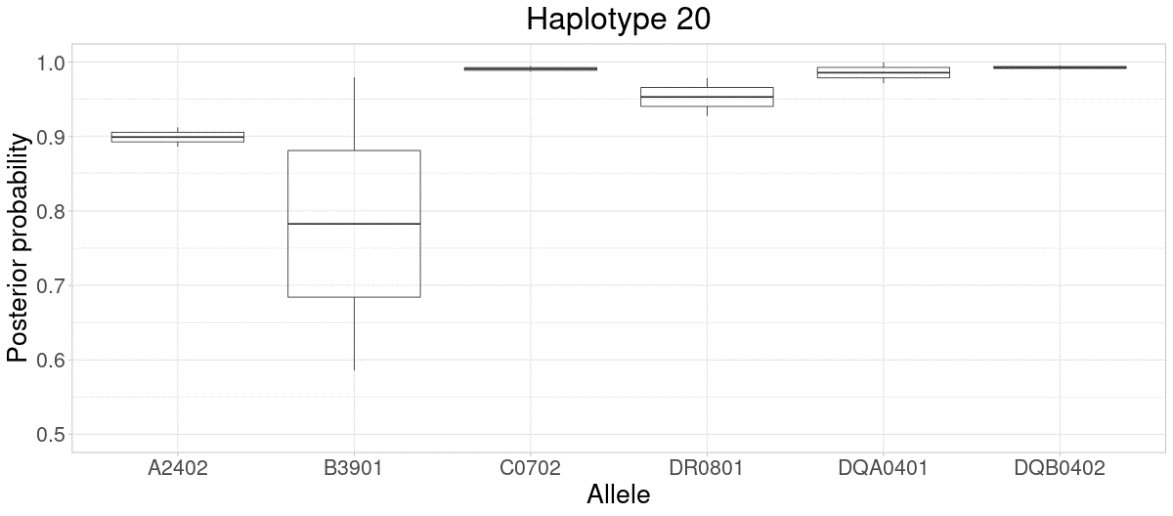


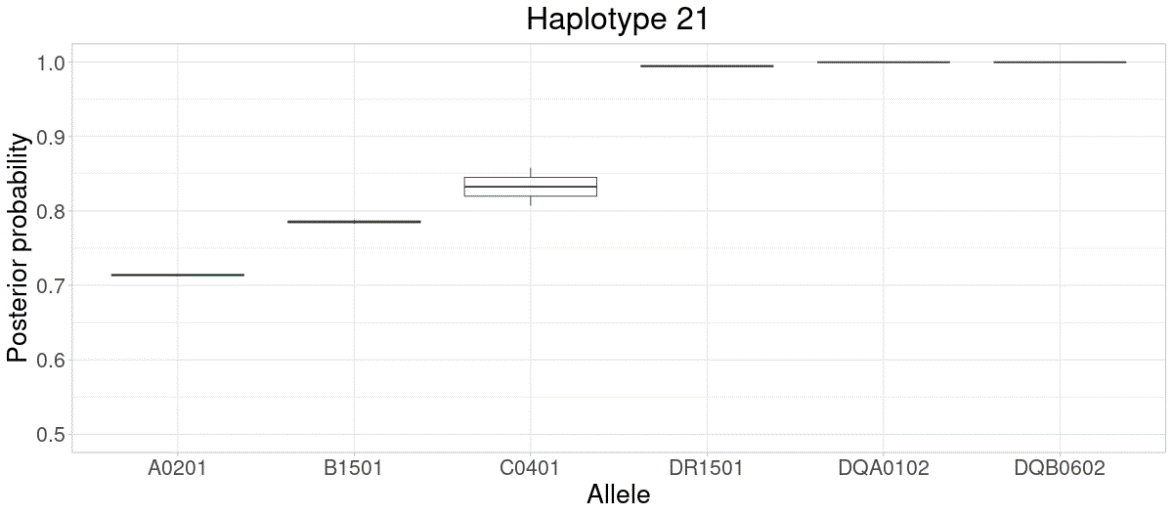

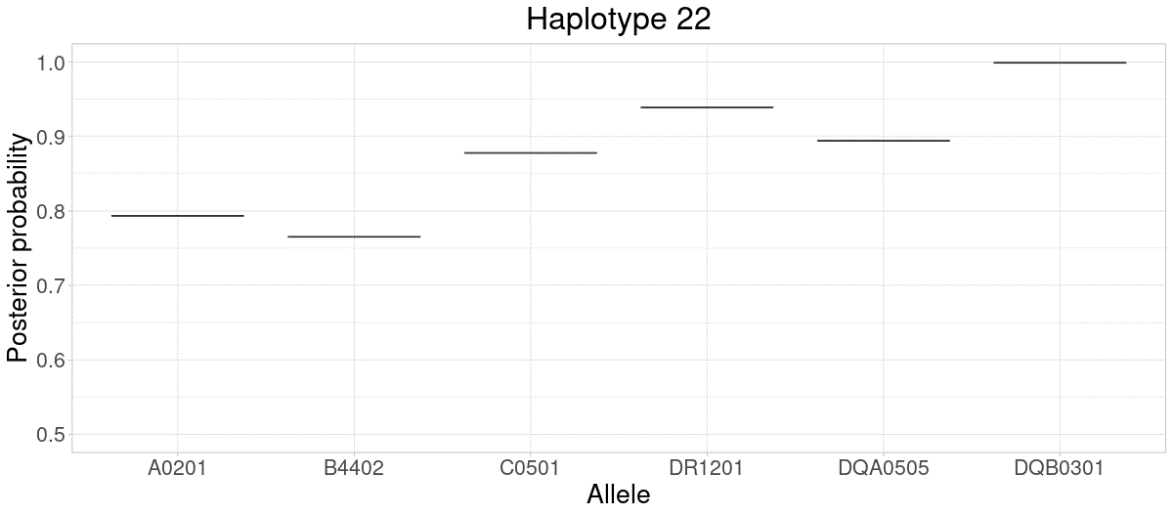


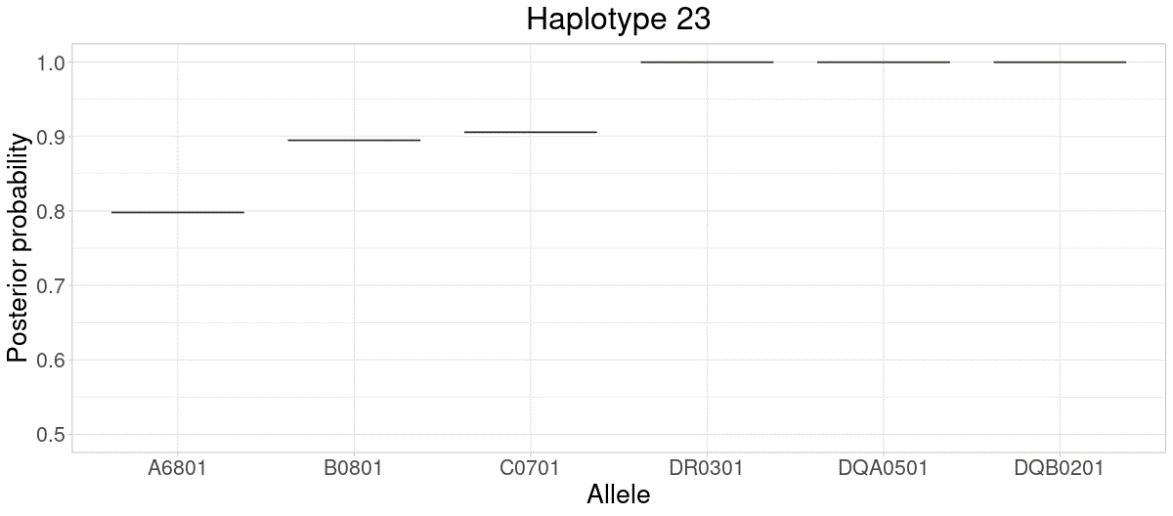

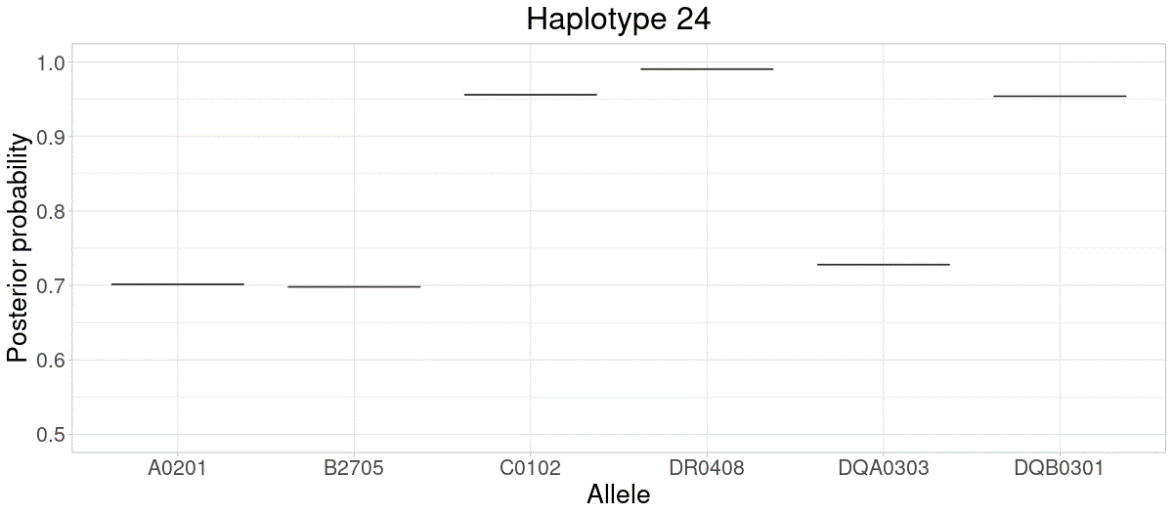


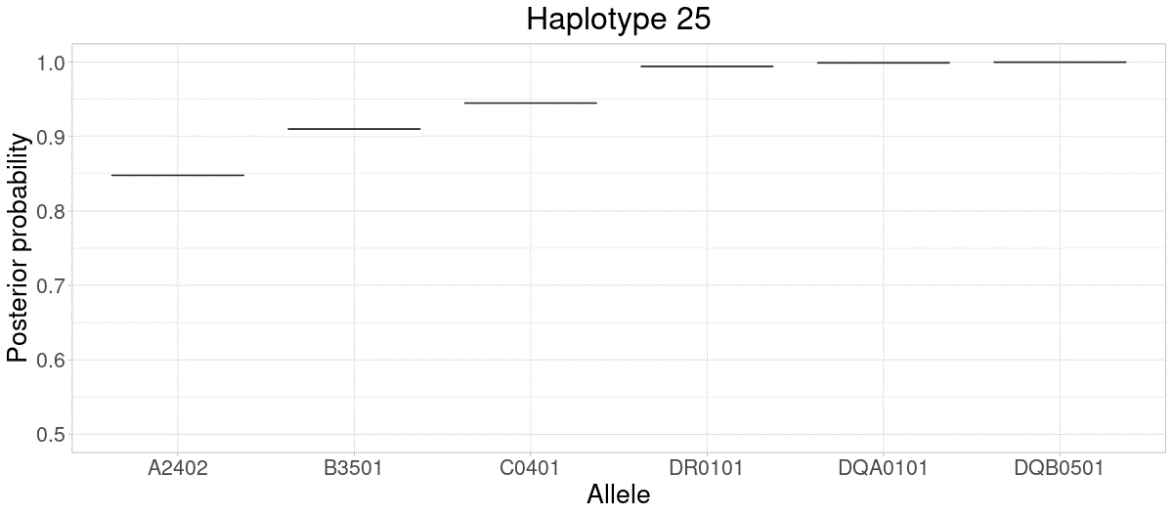

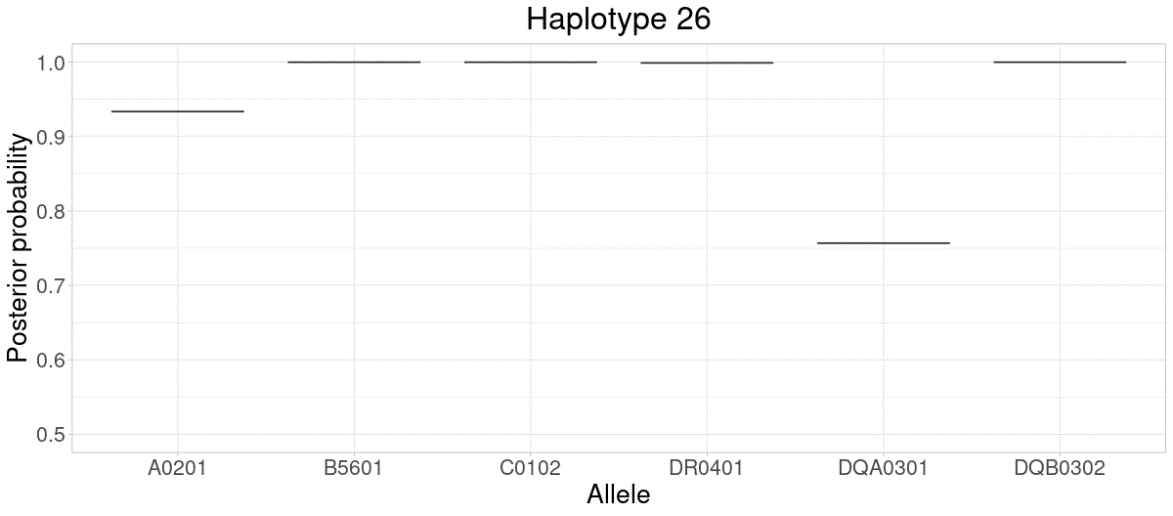


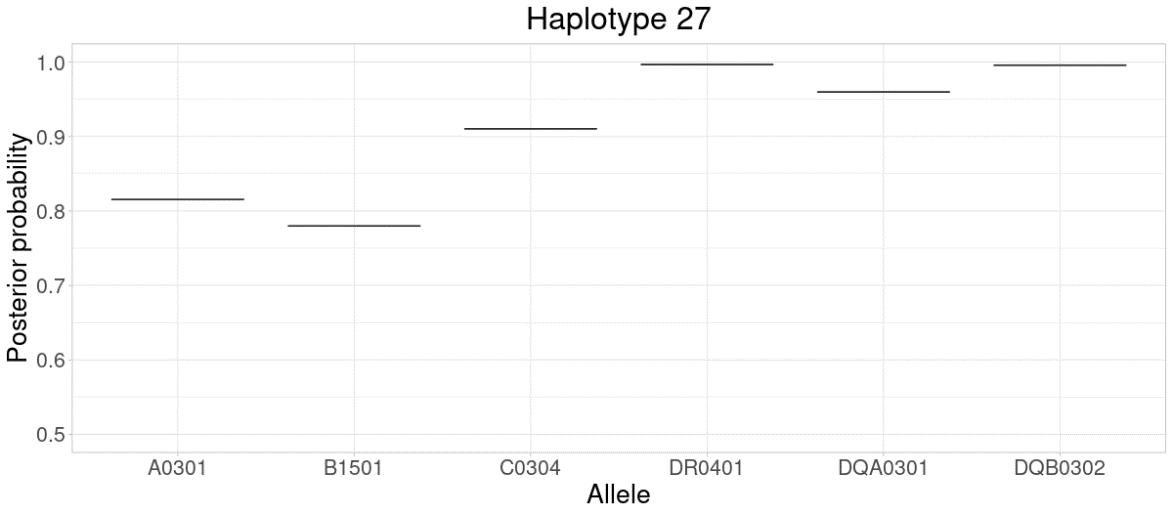

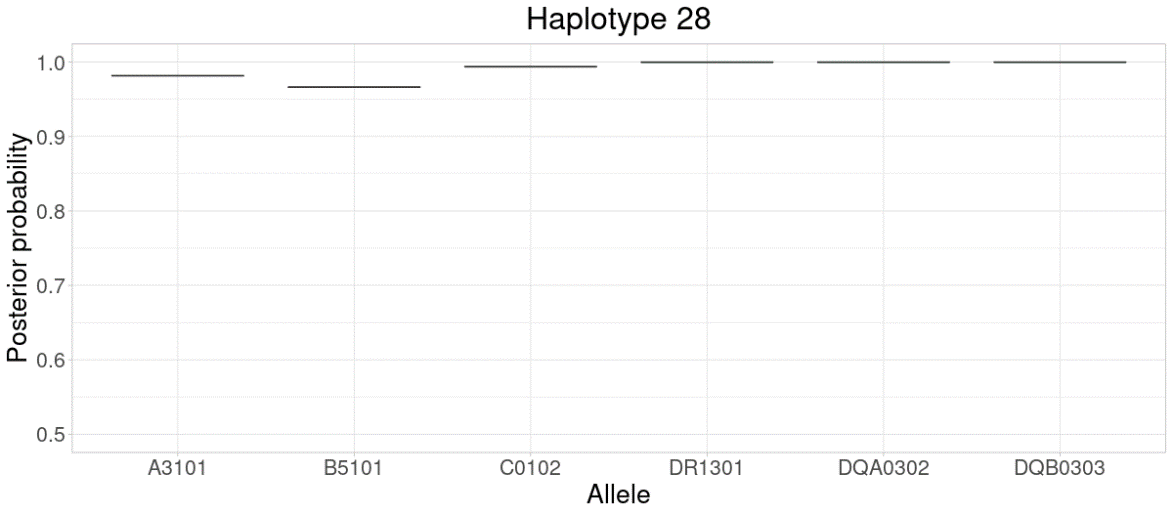


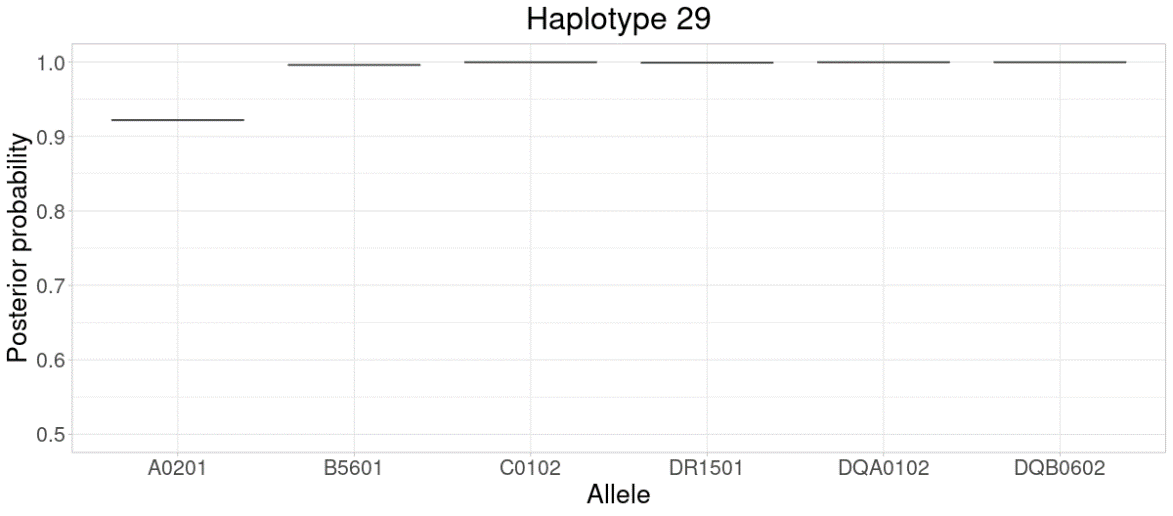

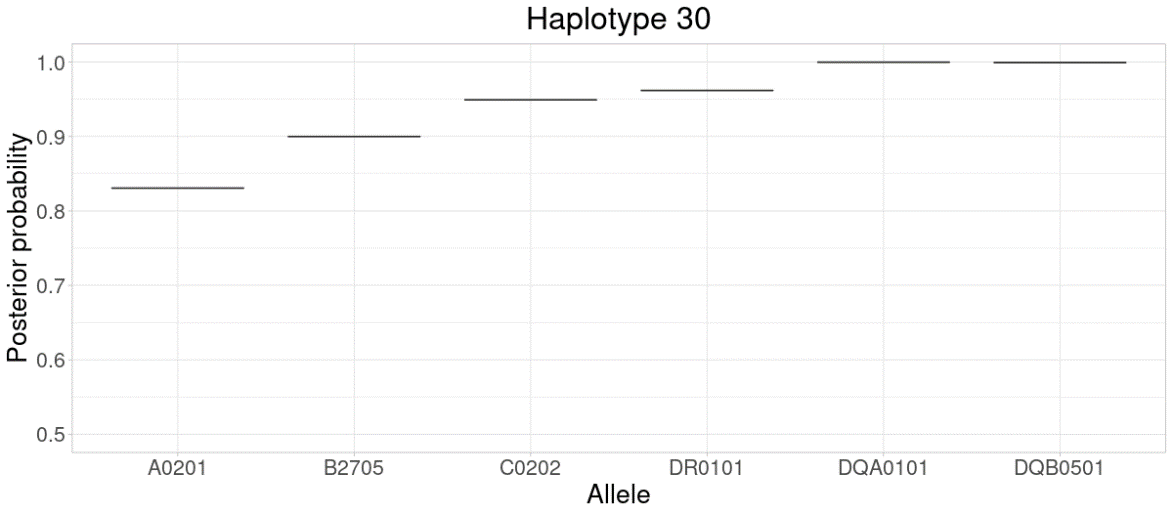


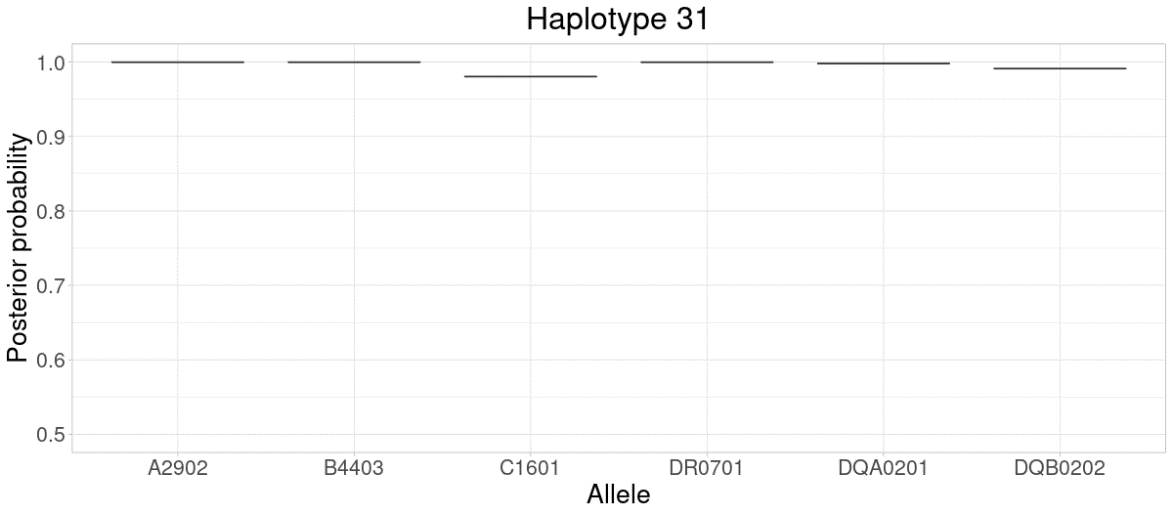

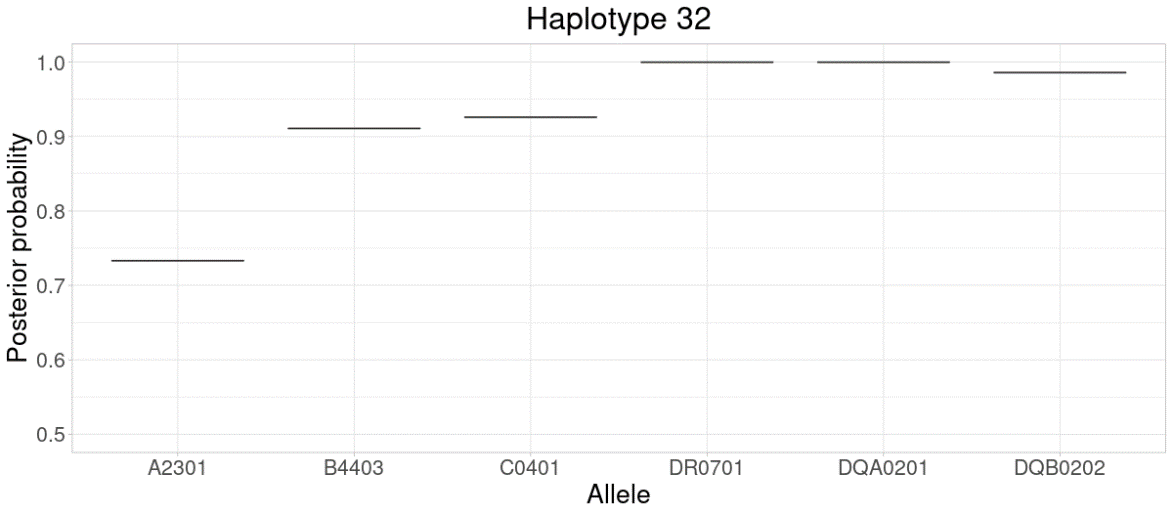


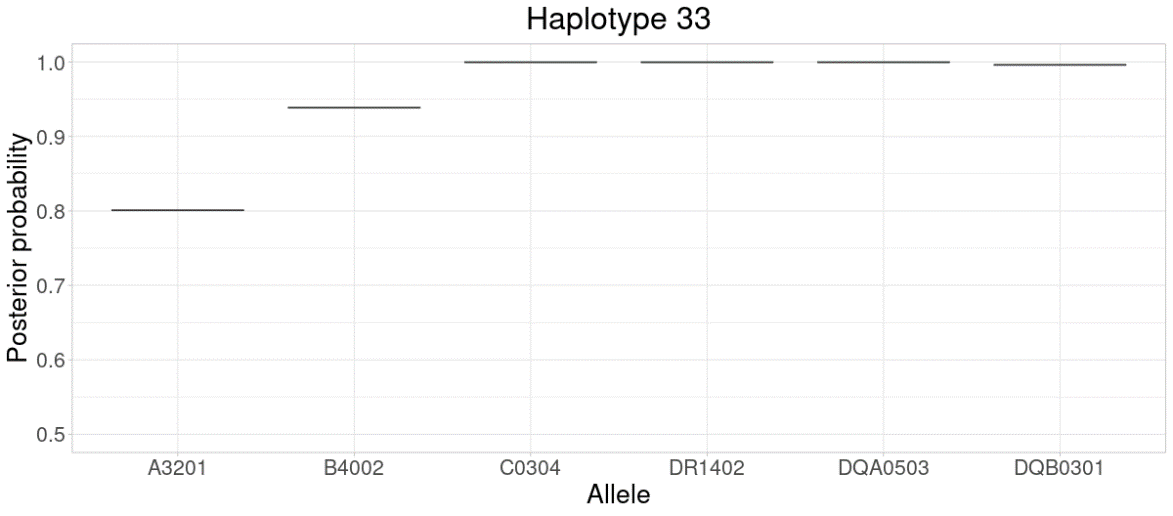

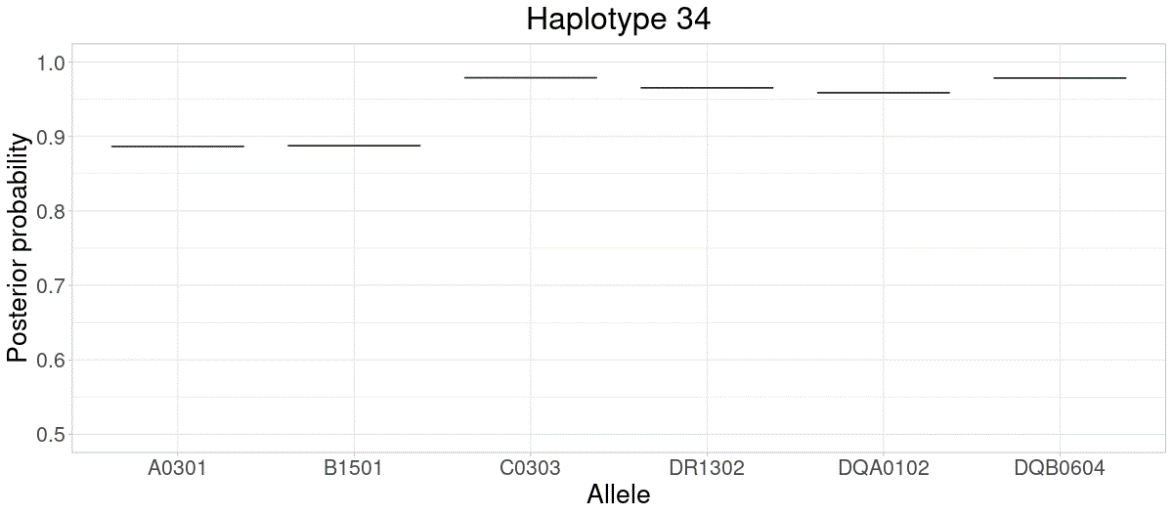


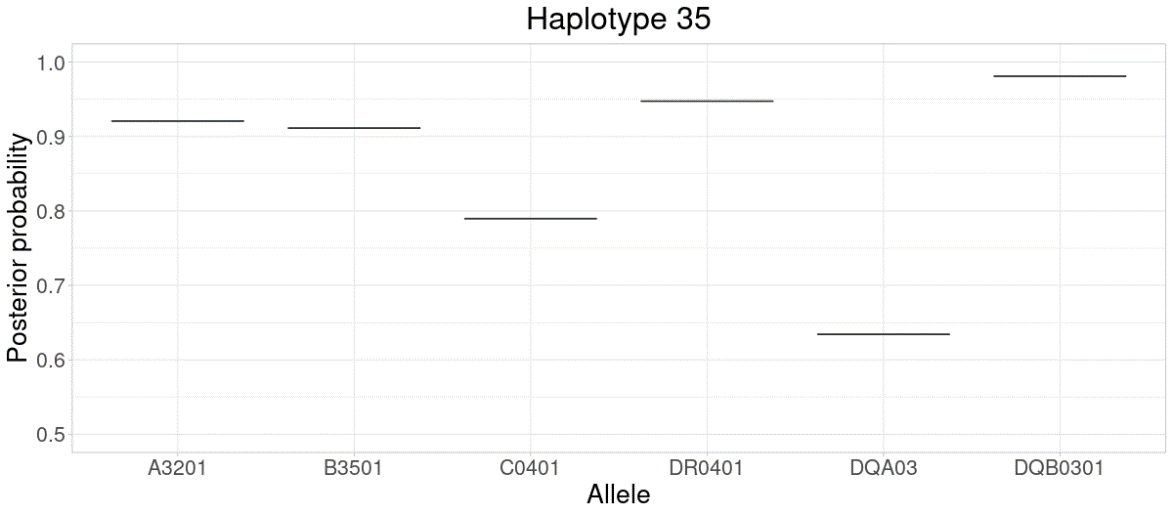

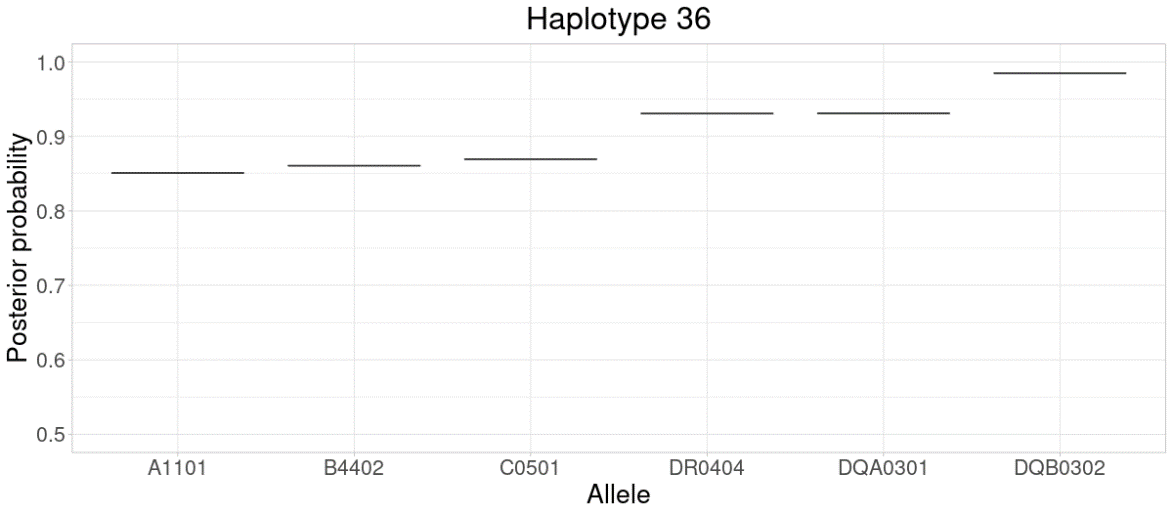


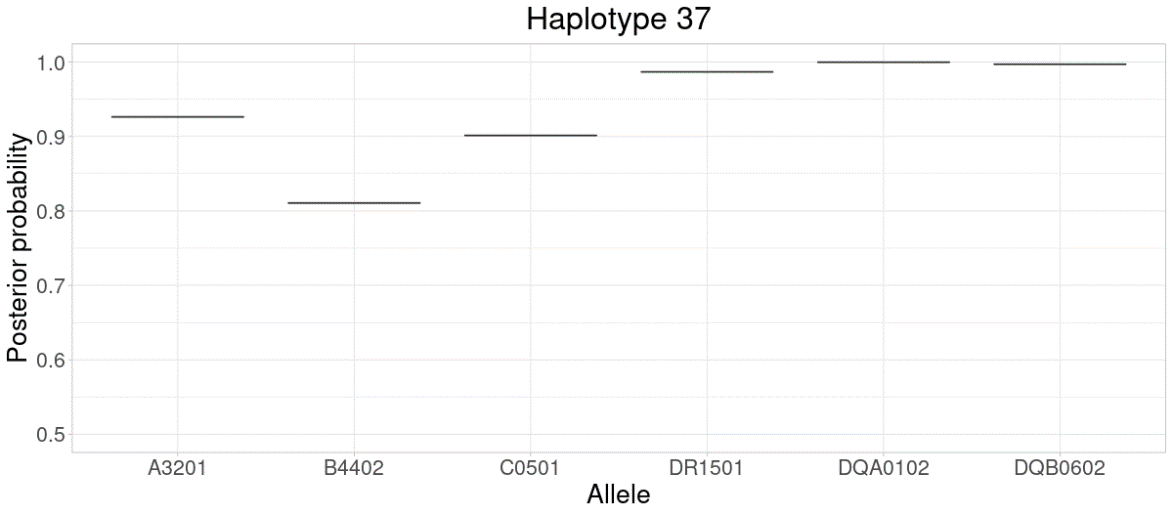

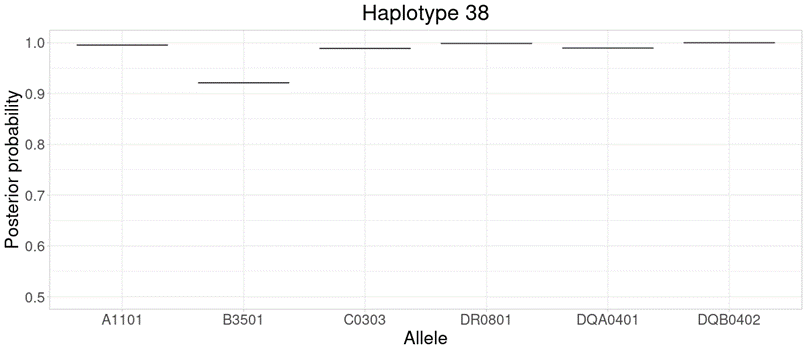


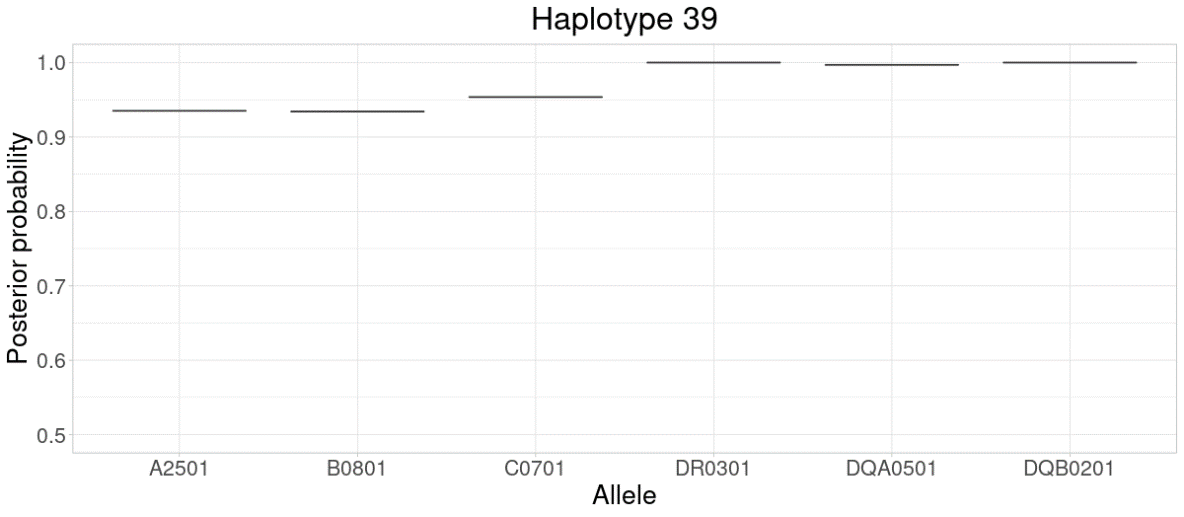

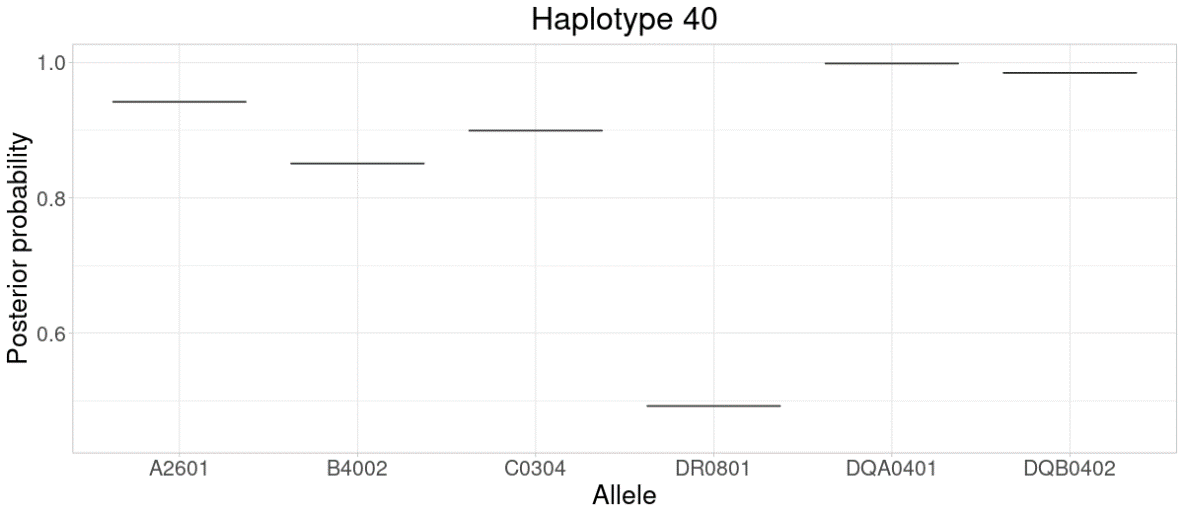


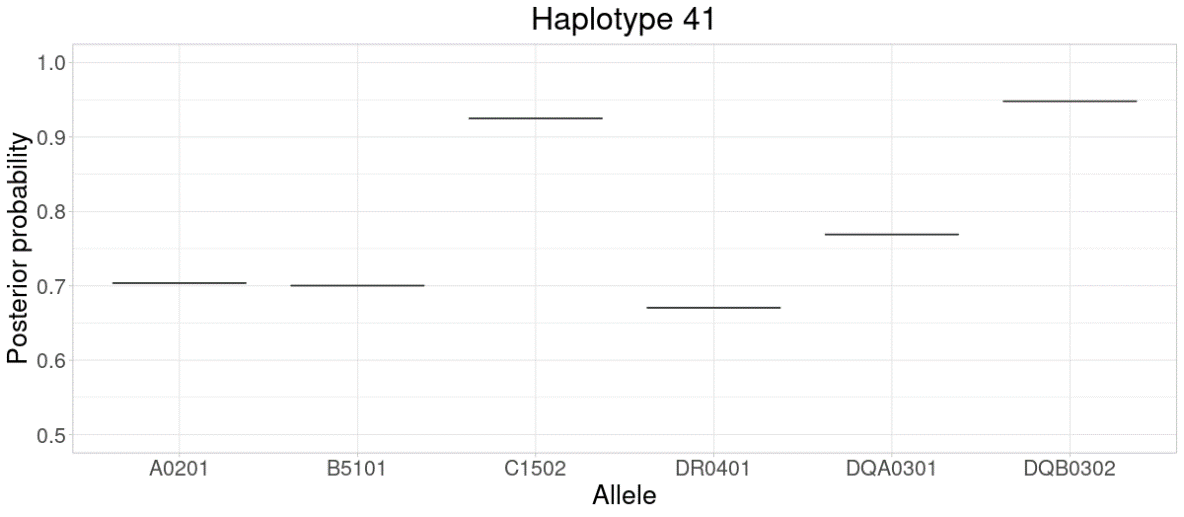

Supplement: Supplementary file 1 — Additional file 1: Figure 1Posterior probabilities of the imputed HLA alleles in a given HLA haplotype. Number of individuals homozygous for each haplotype (1-41) is stated in Table 1. Median, highest and lowest values of posterior probabilities are shown in haplotypes 1-21, and the actual posterior probability value in haplotypes (22-41) where one individual was identified. [file 13287_2022_3182_MOESM1_ESM.docx]
