# Supplementary figures and images for: Blood donor biobank and HLA imputation as a resource for HLA homozygous cells for therapeutic and research use
Source: Stem Cell Res Ther. 2022 Oct 9;13:502. doi: 10.1186/s13287-022-03182-7 (PMC9549658; doi:10.1186/s13287-022-03182-7)

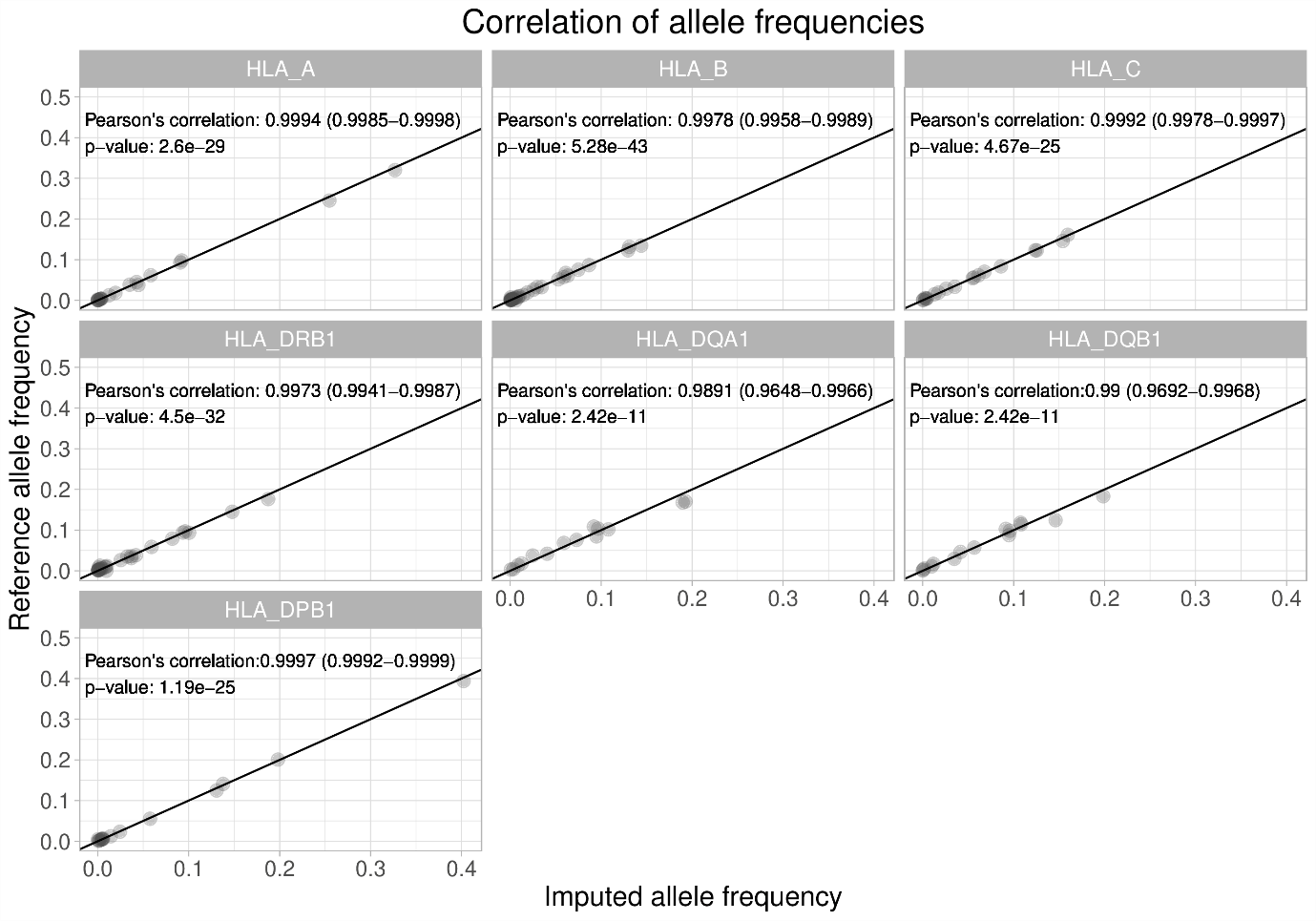

Supplement: Supplementary file 3 — Additional file 3: Figure 3. Correlation analysis of the allele frequencies of the imputed data and the reference data. [file 13287_2022_3182_MOESM3_ESM.docx]
